# A Review of Multiscale Interaction Mechanisms of Wind–Leaf–Droplet Systems in Orchard Spraying

**DOI:** 10.3390/s25154729

**Published:** 2025-07-31

**Authors:** Yunfei Wang, Zhenlei Zhang, Ruohan Shi, Shiqun Dai, Weidong Jia, Mingxiong Ou, Xiang Dong, Mingde Yan

**Affiliations:** 1 School of Agricultural Engineering, Jiangsu University, Zhenjiang 212013, China; 2112416035@stmail.ujs.edu.cn (Y.W.); 2222416001@stmail.ujs.edu.cn (Z.Z.); 2222416009@stmail.ujs.edu.cn (R.S.); jiaweidong@ujs.edu.cn (W.J.); myomx@ujs.edu.cn (M.O.); dongxiang@ujs.edu.cn (X.D.); 2 Key Laboratory of Plant Protection Engineering, Ministry of Agriculture and Rural Affairs, Jiangsu University, Zhenjiang 212013, China; 3 Science Innovation Center, Chinese Academy of Agriculture Mechanization Sciences Group Co., Ltd., Beijing 100083, China; ymd@caams.org.cn

**Keywords:** orchard spraying, key spray variables, wind–leaf–droplet interaction, data-driven modeling

## Abstract

The multiscale interactive system composed of wind, leaves, and droplets serves as a critical dynamic unit in precision orchard spraying. Its coupling mechanisms fundamentally influence pesticide transport pathways, deposition patterns, and drift behavior within crop canopies, forming the foundational basis for achieving intelligent and site-specific spraying operations. This review systematically examines the synergistic dynamics across three hierarchical scales: Droplet–leaf surface wetting and adhesion at the microscale; leaf cluster motion responses at the mesoscale; and the modulation of airflow and spray plume diffusion by canopy architecture at the macroscale. Key variables affecting spray performance—such as wind speed and turbulence structure, leaf biomechanical properties, droplet size and electrostatic characteristics, and spatial canopy heterogeneity—are identified and analyzed. Furthermore, current advances in multiscale modeling approaches and their corresponding experimental validation techniques are critically evaluated, along with their practical boundaries of applicability. Results indicate that while substantial progress has been made at individual scales, significant bottlenecks remain in the integration of cross-scale models, real-time acquisition of critical parameters, and the establishment of high-fidelity experimental platforms. Future research should prioritize the development of unified coupling frameworks, the integration of physics-based and data-driven modeling strategies, and the deployment of multimodal sensing technologies for real-time intelligent spray decision-making. These efforts are expected to provide both theoretical foundations and technological support for advancing precision and intelligent orchard spraying systems.

## 1. Introduction

With the transformation of agricultural production towards greater precision, informatization, and sustainability, orchard pest and disease control are increasingly shifting from traditional, extensive spraying practices to more targeted, site-specific, and eco-friendly approaches [1,2,3,4]. As one of the most widely employed and impactful technologies in orchard protection, spraying systems play a critical role in determining pesticide utilization efficiency and fruit safety [5]. However, conventional spraying methods typically rely on empirically set parameters, often neglecting essential variables such as crop spatial architecture [6,7] and dynamic wind field conditions. This results in substantial pesticide drift [8], poor deposition efficiency, and heightened environmental contamination. In this study, the term “wind” specifically refers to the artificially generated airflow produced by air-assisted spraying systems. This controlled airstream forms a defined wind field within the spraying apparatus, which is designed to optimize droplet trajectory, enhance canopy penetration, and improve deposition efficiency within the canopy structure. In contrast to ambient wind—an uncontrolled environmental factor—the airflow generated by the sprayer constitutes a regulated aerodynamic field that directly influences droplet drift and deposition dynamics.

Under complex canopy structures and fluctuating wind conditions, conventional sprayers often achieve leaf surface deposition rates of less than 30%, leaving a large proportion of droplets suspended in the air or deposited in non-target zones, thereby contributing to off-target drift and aerosol pollution [9]. Such inefficiencies not only escalate operational costs, but also conflict with the overarching goals of green agricultural development and input reduction.

In recent years, emerging technologies such as air-assisted and electrostatic spraying have been increasingly adopted in orchard applications [10,11,12,13,14], showing promise in enhancing droplet penetration and adhesion efficiency. However, their field performance is often constrained by the coupled influence of multiple physical processes. In particular, the dynamic interactions among wind field disturbances, leaf motion, and droplet behavior form a highly integrated, multiscale, and multiphysics system [15,16]. This system is characterized by strong spatiotemporal variability and pronounced non-linear responses, which substantially affect the transport trajectories of spray clouds, the spatial distribution of droplet deposition, and the overall effectiveness of pesticide adherence.

Specifically, wind acts as an external excitation that not only governs the transport behavior of droplets, but also induces complex responses in plant leaves, including vibration, deformation, and torsion [17]. The dynamic motion of flexible leaves under wind excitation can significantly reshape the local airflow structure and alter droplet trajectories [18]. When leaves undergo rapid oscillations, their ability to intercept droplets diminishes, resulting in a lower deposition probability. In contrast, more stable leaf surfaces serve as effective targets, enhancing droplet capture efficiency. Moreover, the fate of a droplet upon impact—whether it deposits, rebounds, or shatters—is regulated by microscale physical attributes such as surface wettability, roughness, and contact angle [19,20]. These properties are not static; they evolve dynamically with leaf motion, amplifying the degree of coupling within the system.

From a multiscale analytical perspective, the wind–leaf–droplet interaction system can be divided into three hierarchical levels. At the microscale, the focus lies on droplet–leaf surface interactions, including wetting, adhesion, and evaporation behaviors [21]. The mesoscale emphasizes coordinated movements and occlusion dynamics among leaf clusters [22]. At the macroscale, the structure of the wind field, ventilation pathways within the canopy, and the reconstruction of the overall flow regime are considered [23]. This nested, feedback-driven multiscale system poses significant challenges for conventional modeling and optimization approaches that rely on static assumptions or empirical parameter tuning, rendering them inadequate for capturing the spatiotemporal complexity of real orchard environments.

Although considerable progress has been made in modeling droplet transport, analyzing flexible leaf responses, and predicting localized deposition behavior, most existing studies isolate these processes and lack a unified framework that captures the dynamic coupling among wind, leaves, and droplets. Current modeling strategies face substantial technical barriers in terms of multiscale coupling representation, parameter harmonization, simulation fidelity, and experimental validation. These limitations hinder the translation of research findings into practical applications for intelligent and high-efficiency orchard spraying systems.

Based on the above, this review aims to systematically examine the multiscale interaction mechanisms within the wind–leaf–droplet ternary system, with the objective of identifying the key physical variables influencing spray quality, summarizing recent advances in multiscale modeling strategies and experimental observation techniques, and analyzing the core challenges and research gaps in system integration. Furthermore, this work seeks to establish a logically coherent research framework that links microscale, mesoscale, and macroscale mechanisms through a unified perspective, thereby enhancing the overall understanding of the coupled dynamics within the ternary system.

The specific objectives of this review include the following:

1. Elucidating the coupling pathways and physical mechanisms between wind fields, leaf dynamics, and droplet behavior across multiple spatial and temporal scales;
2. Identifying the key variables that affect orchard spray performance and droplet deposition efficiency, along with their modes of influence;
3. Reviewing the state-of-the-art modeling approaches and visualization techniques currently used for spray system analysis, along with their applicability and limitations;
4. Analyzing existing challenges such as insufficient model integration, difficulties in parameter acquisition, and the lack of high-resolution experimental validation;
5. Proposing future research directions and potential technological breakthroughs to support the development of responsive, environmentally adaptive, and flexibly deployable intelligent spraying systems.

Through these efforts, this review aspires to advance the study of orchard spraying systems from isolated process analyses toward mechanism-driven, multiscale modeling paradigms, thereby contributing a theoretical foundation for the development of efficient, sustainable, and intelligent plant protection technologies in orchard environments.

## 2. Multiscale Coupling Mechanisms of Wind–Leaf–Droplet Interactions

### 2.1. Microscale Interactions: Droplet–Leaf Surface Dynamics

In orchard spraying, microscale interactions between droplets and leaf surfaces are critical in determining pesticide deposition efficiency and utilization. This process involves complex multiphysics mechanisms—including droplet dynamics, wetting behavior, and microstructural properties of the leaf surface—constituting a typical multiscale, multi-factor coupled system. Upon impact with the leaf surface, droplets may undergo deformation, spreading, rebound, coalescence, fragmentation, or evaporation, each of which directly affects pesticide retention and bioavailability.

**Figure 1:**
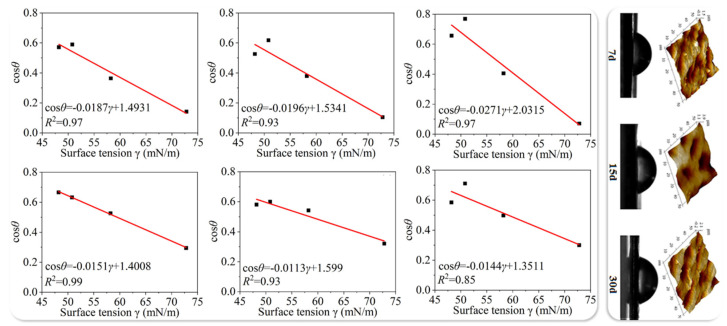
Contact angle–surface tension relationships and microstructural features on the adaxial and abaxial surfaces of mango leaves: This figure illustrates the fitted contact angle curves and corresponding microstructural characteristics of both adaxial and abaxial leaf surfaces at different growth stages, subjected to four types of test solutions. The results indicate an overall increasing trend in contact angle with leaf maturation, accompanied by a well-defined variation in critical surface tension. The Zisman fitting curves reveal the responsiveness of droplet spreading behavior to changes in surface energy. Contact angle measurements and atomic force microscopy (AFM) images reveal the evolution of surface roughness and microstructure, highlighting how these structural changes collectively influence the wetting behavior of mango leaves.

The initial behavior of spray droplets is primarily governed by their kinetic energy and surface tension [24], and is commonly characterized using dimensionless parameters such as the Weber number (We), Reynolds number (Re), and contact angle. A higher Weber number generally indicates a greater tendency for droplet deformation or breakup upon impact [25,26,27], while the contact angle determines the spreading capacity of droplets on the leaf surface [28], as illustrated in Figure 1. Hydrophilic surfaces facilitate film-like deposition, promoting effective pesticide retention, whereas hydrophobic or wax-coated surfaces tend to induce droplet rebound or shattering [29], thereby reducing the overall utilization efficiency of the pesticide.

**Figure 2:**
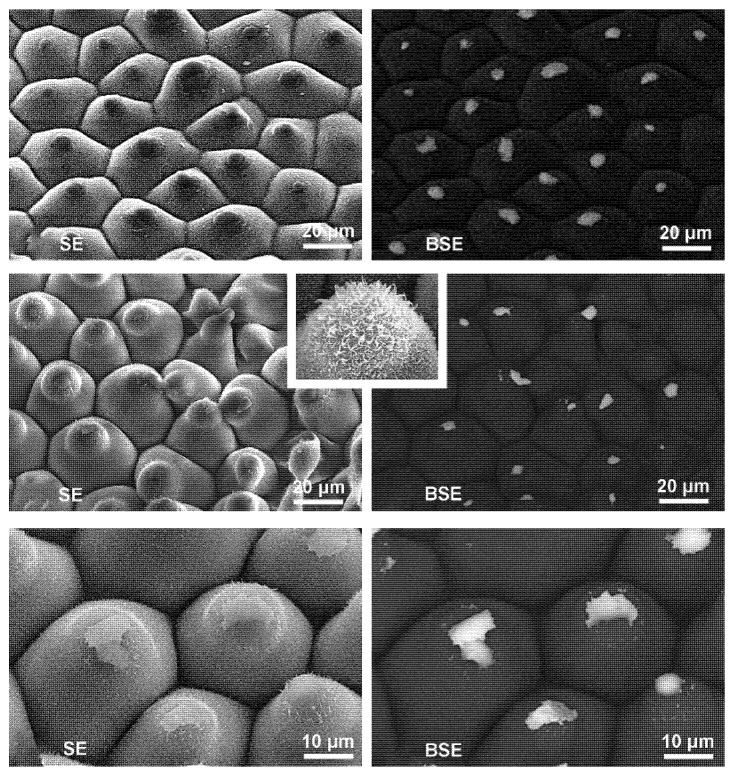
Droplet residue and microstructural changes on superhydrophobic leaf surfaces: Microscopic images illustrate the effects of mechanical abrasion and droplet impact on the surface structure and hydrophobicity of lotus and colocasia leaves. Light wiping partially damages the wax crystals at protruding regions, leading to droplet residue, while droplet impact causes localized structural disruption and liquid retention within surface grooves.

The structural properties of the leaf surface play a pivotal role in regulating droplet behavior. Many fruit tree leaves exhibit micro- to nanoscale roughness and possess waxy layers, which significantly influence wetting modes and droplet adhesion stability [30]. Studies have shown that superhydrophobic surfaces tend to induce more pronounced droplet rebound, while smoother or hydrophilic-modified surfaces facilitate droplet spreading and retention. Recent advances using Scanning Electron Microscopy (SEM) and Atomic Force Microscopy (AFM) have enabled detailed investigations into the relationship between leaf surface microstructure and droplet dynamics [31], as illustrated in Figure 2.

In practical orchard operations, leaves are frequently subjected to wind-induced vibrations, and their dynamic response can significantly alter the angle of droplet impact and relative velocity [32]. When the direction of leaf motion aligns with the incoming droplet stream, the likelihood of droplet adhesion increases; conversely, opposing motion tends to promote slippage or rebound. Experimental studies have confirmed that leaf acceleration, oscillation frequency, and phase angle notably influence microscale deposition efficiency [33,34], necessitating their consideration in spraying strategy design.

Ambient temperature and humidity also influence droplet behavior. Fine droplets are particularly susceptible to rapid evaporation under high-temperature and low-humidity conditions, leading to substantial pesticide loss [35,36,37]. To address this, some studies have proposed thermo–mass coupling models to simulate droplet evaporation processes [38], with validation through infrared thermography and related techniques, aiding in the optimization of spraying time windows and environmental adaptability.

In addition, the composition of the spray formulation—especially the inclusion of surfactants and adjuvants—plays a critical role in modulating microscale droplet–leaf interactions [39,40,41]. At low concentrations, surfactants reduce surface tension and improve wetting behavior; however, excessive concentrations may trigger demulsification, coalescence, or abrupt shifts in wetting modes, thereby compromising deposition stability. Hence, spray formulation is a key variable governing droplet behavior and deposition at the leaf interface. Table 1 summarizes representative studies and key findings on the effects of surfactants and adjuvants on droplet–leaf surface interactions.

To elucidate these microscale mechanisms, several experimental and computational approaches have been widely adopted. High-speed imaging is employed to analyze droplet impact dynamics; laser-induced fluorescence (LIF) techniques are used to evaluate droplet distribution and surface coverage; contact angle measurements quantify surface wettability; and numerical simulation methods such as Volume of Fluid (VOF) and Smoothed Particle Hydrodynamics (SPH) are utilized to construct microscale models and conduct sensitivity analyses on key parameters.

Overall, although microscale processes are localized in nature, their effects accumulate at the macroscale, ultimately influencing the resource-use efficiency and environmental sustainability of the spraying system. Future spray system design should place greater emphasis on the integrated modeling of leaf surface heterogeneity, dynamic leaf motion, and droplet formulation properties. Such efforts would facilitate a methodological shift from empirically tuned parameters to mechanism-driven optimization, advancing the development of intelligent and environmentally adaptive spraying technologies.

### 2.2. Mesoscale Dynamics: Local Leaf Motion and Coordinated Effects

In orchard environments, plant leaves are often arranged in clustered formations, constituting localized canopy systems that exhibit collective dynamic responses under wind disturbances, such as synchronized swaying, phase-shifted oscillations, and overlapping displacements [47,48]. This mesoscale coordinated response bridges the gap between the microscale vibration of individual leaves and the macroscale deformation of the entire canopy, serving as a critical link for understanding the multiscale coupling mechanisms among wind, leaves, and droplets. Studies have shown that varying wind speeds and gust conditions can induce either synchronous or asynchronous motion patterns among leaf clusters [49]. The former tends to form stable occlusion structures, thereby enhancing the uniformity of droplet deposition. In contrast, asynchronous responses often lead to frequent opening–closing transitions, increasing the risk of off-target spraying.

**Figure 3:**
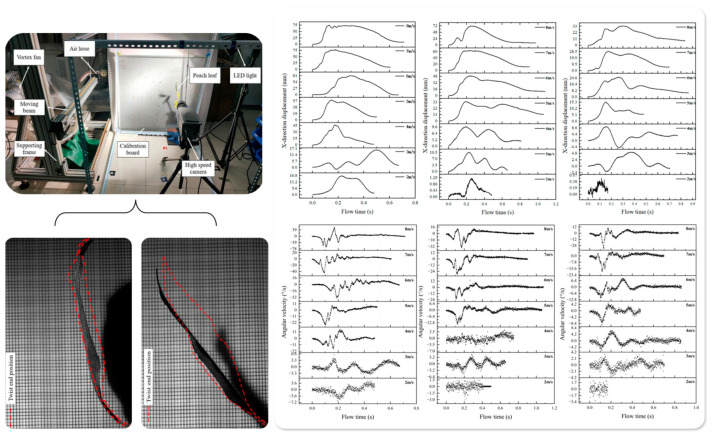
Measurement system and analysis results of peach leaf motion characteristics based on high-speed photography and image tracking: The top-left panel illustrates the experimental setup, which includes a vortex fan, high-speed camera, LED lighting, calibration board, and leaf clamping device. The bottom-left panel shows the schematic diagram of leaf posture changes during the torsional motion. The right panels present the time-series variations of lateral displacement, vertical displacement, and angular velocity of the leaf tip under different wind speeds (2–8 m/s) and platform translation speeds (0.5, 0.75, and 1.0 m/s). The results indicate that both wind speed and platform movement significantly affect the leaf’s oscillation amplitude, torsional duration, and dynamic response characteristics.

Recent research has employed high-speed imaging and motion-tracking techniques [50,51,52] to capture time-series data of leaf cluster movements, as illustrated in Figure 3. By applying Fourier transform and spectral analysis, key parameters such as dominant frequency, oscillation modes, and phase differences have been extracted [53], enabling a quantitative characterization of the relationship between collective leaf dynamics and droplet deposition efficiency.

In addition to structural responses, significant aerodynamic coupling effects also exist among adjacent leaves within a cluster. When airflow passes through narrow gaps in dense leaf groups, it often induces complex flow phenomena such as vortices, wake disturbances, and localized pressure differentials. These unsteady aerodynamic structures, in turn, exert feedback forces on downstream leaves, establishing a characteristic fluid–structure interaction (FSI) mechanism [54]. Studies have shown that such coordinated leaf motion can periodically open or close “dynamic channels” within the canopy during spraying operations, thereby regulating the penetration pathways and deposition behaviors of spray droplets [55]. When the leaf cluster is in an open configuration, droplets can pass through and reach deeper canopy layers. Conversely, during the closing phase, droplets may be obstructed, deflected, or reflected, resulting in increased drift or surface accumulation.

Notably, droplet behavior within these dynamic channels exhibits a strong dependency on droplet size. Fine droplets are more likely to follow the airflow and penetrate through the transient openings, whereas larger droplets tend to deposit on outer leaf surfaces or be bounced back, highlighting a dynamic size-matching effect between droplet diameter and canopy motion.

**Figure 4:**
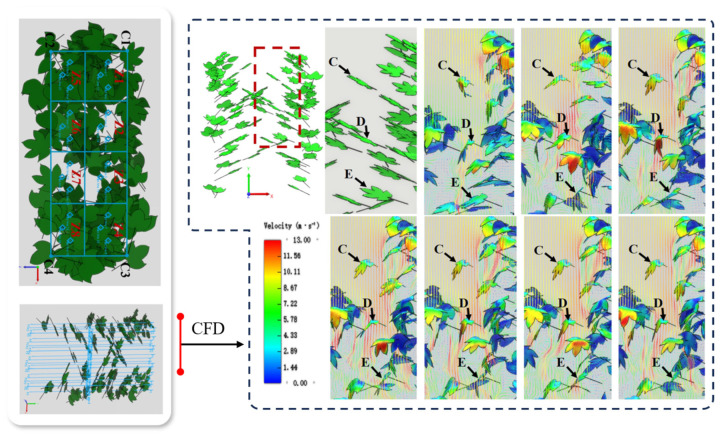
Dynamic response of canopy leaves and flow field coupling under wind loading: The figure illustrates the coupled airflow–leaf interaction simulated using a CFD-based model. The canopy environment was simulated using the Reynolds-averaged Navier–Stokes (RANS) equations with the standard k–ε turbulence model, assuming incompressible and steady-state flow. The leaf surfaces were modeled as rigid and non-deformable. Boundary conditions included a uniform inlet velocity of 12 m/s, a pressure outlet, and no-slip wall conditions. Simulation results show that exposed leaves experience strong aerodynamic responses, with distinct vortex formation observed on the leeward side. In contrast, leaf clusters exhibit relatively weaker motion. Under continuous airflow, leaf vibration gradually stabilizes, and low-velocity channels emerge within the canopy, potentially enhancing droplet penetration. The color map represents airflow velocity, with red indicating high-speed regions and blue representing low-speed zones. Leaves labeled C, D, and E represent three typical leaves at different positions in the canopy used to analyze their deformation and interaction with the airflow over time.

From a modeling perspective, previous studies have employed lumped parameter models or flexible continuum models coupled with computational fluid dynamics (CFD) platforms to simulate the dynamic responses of local leaf clusters under wind field conditions [56], as illustrated in Figure 4. Some works have further integrated discrete element methods (DEMs) and multibody flexible dynamics approaches to model the coordinated motion of multiple leaves subjected to complex airflow interactions. The simulated leaf dynamics and local flow field structures revealed through these models offer valuable insights for identifying spray windows and controlling nozzle activation timing. Such information supports the development of variable-rate spraying strategies, particularly for optimizing targeted pesticide application in multilayered canopy environments.

Despite ongoing advancements, significant challenges remain at the mesoscale level. First, the high heterogeneity of leaf cluster structures—resulting from variations in species, growth stages, and management practices—limits the generalizability of existing models. Second, experimental data acquisition under natural conditions is hindered by factors such as lighting interference, spatial occlusion, and tracking inaccuracies, making it difficult to conduct long-term, stable measurements of coordinated leaf motion. Third, the system-level integration of mesoscale structural responses with droplet transport processes remains underdeveloped, preventing a comprehensive quantification of their effects on deposition patterns.

In summary, the coordinated response of leaf clusters at the mesoscale serves as a critical mediating variable in the wind–leaf–droplet coupling mechanism. It plays a pivotal role in dynamically regulating canopy ventilation channels, shaping droplet penetration windows, and influencing the spatial distribution of pesticide deposition. A deeper understanding of its dynamic behavior will support the development of adaptive spray control strategies based on local structural responses, thereby enhancing the intelligence and precision of orchard spraying systems.

### 2.3. Macroscale Structure: Canopy Ventilation and Spray Cloud Distribution

Macroscale studies focus on the interaction between airflow and orchard canopy structure, and how this interaction reshapes air movement pathways and influences the overall transport and deposition patterns of spray clouds. As the outermost scale of the wind–leaf–droplet system, the macroscale layer determines spray uniformity, penetration capability, and operational coverage, serving as the physical foundation for the design of precision spraying strategies.

**Figure 5:**
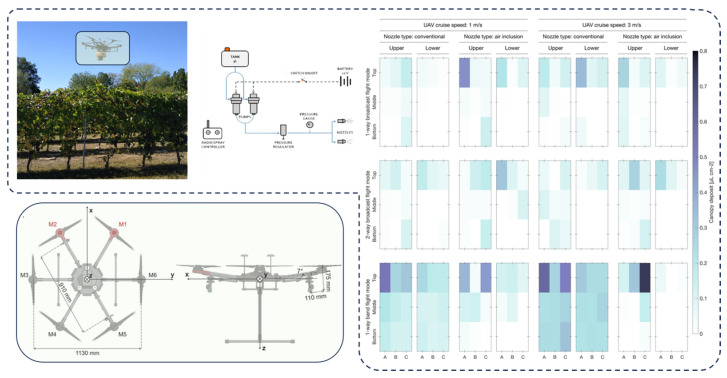
Effects of grapevine canopy structure on spray deposition: This figure presents the droplet deposition distribution across different vertical canopy layers under varying spray patterns and canopy structural conditions. The experimental results indicate that the upper canopy consistently receives significantly higher droplet deposition compared to the middle and lower layers. This discrepancy is particularly pronounced under the broadcast flying mode, highlighting the issue of insufficient penetration commonly associated with conventional spraying. In contrast, the use of a unidirectional strip-flying mode combined with standard nozzles substantially improves deposition uniformity in the middle and lower canopy regions. Although variations in canopy depth (A, B, and C) did not yield a consistent trend, the overall findings suggest that the vertical heterogeneity of the canopy plays a dominant role in regulating droplet deposition efficiency and spatial distribution. This figure underscores the critical influence of canopy structural heterogeneity on spray performance.

Orchard canopies exhibit pronounced spatial heterogeneity [57]. Variations in canopy density, tree height, branch orientation, and leaf inclination across species significantly affect canopy porosity and wind resistance [58,59]. These structural differences influence local wind speed, turbulence intensity, and pressure fields, thereby indirectly modulating droplet trajectories and deposition outcomes [60], as illustrated in Figure 5. Among the key parameters governing wind–droplet interactions at this scale are canopy porosity, leaf area index (LAI) [61], vertical stratification, and the spatial configuration of branch flow channels. While higher LAI values tend to enhance droplet interception, overly dense canopy structures may obstruct coverage of interior leaf layers, resulting in an uneven distribution pattern characterized by over-deposition on outer surfaces and under-deposition within the canopy interior.

As airflow passes through the canopy structure, it undergoes velocity attenuation and streamwise reconstruction. Localized regions—such as branch intersections and dense leaf clusters—tend to induce turbulence, wake zones, and vortex structures, which destabilize droplet transport and may lead to retention, rebound, or lateral drift phenomena. These effects collectively impact the timing and spatial accuracy of droplet deposition [62,63].

**Figure 6:**
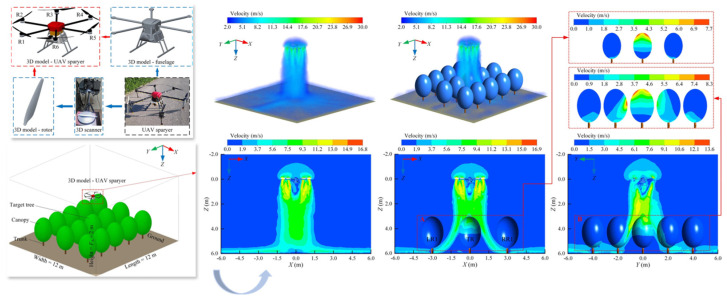
CFD-based analysis of the interaction between UAV downwash airflow and orchard canopy structure: This figure illustrates the regulatory effect of orchard canopy architecture on the downwash airflow generated by a UAV sprayer. The simulation was conducted using the Fluent platform, with a three-dimensional steady-state incompressible flow model and the standard k–ε turbulence model. The initial outlet velocity was set to 15 m/s at a spraying height of 2.5 m. The ground was defined as a no-slip wall, while all other boundaries were treated as pressure outlets. The results indicate that the canopy substantially attenuates the downward airflow, forming a near-zero velocity zone close to the ground and redirecting the flow laterally along the surface. In contrast, under canopy-free conditions, the airflow exhibits a concentrated vertical jet pattern with rapid ground impact and radial dispersion. Cross-sectional views from different directions further demonstrate how canopy structures influence airflow penetration paths and velocity gradients, validating the critical role of canopy architecture in regulating spray penetration and deposition performance.

To quantitatively assess these aerodynamic influences, computational fluid dynamics (CFD) has emerged as a fundamental tool for analyzing the coupled interactions among wind, canopy, and spray droplets. By incorporating realistic canopy parameters—such as leaf area index (LAI) distribution, branch density, and leaf inclination angles—three-dimensional models can be constructed to reflect field-representative conditions. Some studies have further integrated large eddy simulation (LES) approaches to resolve transient airflow structures and reveal the spatial patterns of deposition hotspots and blind zones within the canopy [64], as illustrated in Figure 6.

In addition to simulation-based approaches, field observation technologies have advanced considerably. Tools such as wind profile sensors, infrared imaging, and machine vision combined with deep learning algorithms provide powerful visual support for validating macroscale airflow patterns and detecting canopy structures [65,66,67]. Some studies have leveraged canopy and target detection results to formulate variable-rate spraying strategies, enabling differentiated pesticide application across canopy regions (e.g., upper, lateral, and lower layers) [68], thereby optimizing pesticide utilization and enhancing targeted deposition efficiency.

Moreover, the motion and deformation behavior of spray clouds at the canopy periphery plays a critical role. Under the influence of wind disturbances, thermal boundary effects, or gradients in temperature and humidity, spray clouds may undergo diffusion, deflection, fragmentation, or agglomeration [69], particularly in orchards with wide row spacing or multilayer canopy structures. These large-scale disturbances significantly affect droplet trajectories and the final deposition zones.

Despite continuous advancements in macroscale modeling and observation, two major challenges persist. First, the seasonal dynamics of canopy structure result in substantial temporal variability of model parameters—from sparse spring foliage to dense summer canopies—fundamentally altering airflow channel properties. Second, natural wind fields exhibit high uncertainty; their pulsation characteristics and directional variability are strongly influenced by terrain, shelterbelts, and atmospheric boundary layer conditions, thereby increasing modeling errors and complicating spray control.

Therefore, a comprehensive understanding of how canopy architecture regulates wind–droplet interactions at the macroscale is essential for enabling three-stage spray optimization: source prediction, in-process guidance, and terminal control. Future research should integrate real-time sensing systems for dynamic canopy monitoring and couple these with spray modeling and actuator control frameworks. Such integration will be key to achieving structurally informed, deep-targeted spraying and promoting green, precision-oriented crop protection.

## 3. Key Factors Influencing Spray Quality in Orchards

### 3.1. Wind Speed and Turbulence Structure

Wind speed and turbulence structure are critical environmental factors that determine droplet transport behavior and spray deposition efficiency. These variables not only influence the ability of droplets to penetrate the canopy and reach target leaf surfaces, but also define the overall performance of the spraying system in terms of precision, resource utilization, and environmental safety.

Moderate wind speeds can enhance droplet penetration by enabling droplets to overcome leaf surface obstruction and gravitational settling, thereby facilitating their entry into the middle and lower canopy layers. Under such conditions, a dynamic balance is established between aerodynamic drag and droplet weight, particularly benefiting medium-sized droplets with moderate Stokes numbers. These droplets exhibit favorable inertial response characteristics, allowing them to follow curved airflow paths and effectively traverse canopy gaps.

However, when wind speed becomes excessively high, the relative velocity between droplets and the surrounding airflow increases substantially. As a result, fine droplets become dominated by aerodynamic drag forces, causing them to drift passively with the airflow [70,71,72,73]. Although coarse droplets retain greater inertia, they are more susceptible to perturbations by turbulent structures, often leading to deflection, rebound, or premature settling. Empirical studies have shown that when wind speed exceeds 4.0 m/s, lateral drift significantly increases, leading to reduced deposition efficiency and a markedly higher risk of off-target contamination [74].

**Figure 7:**
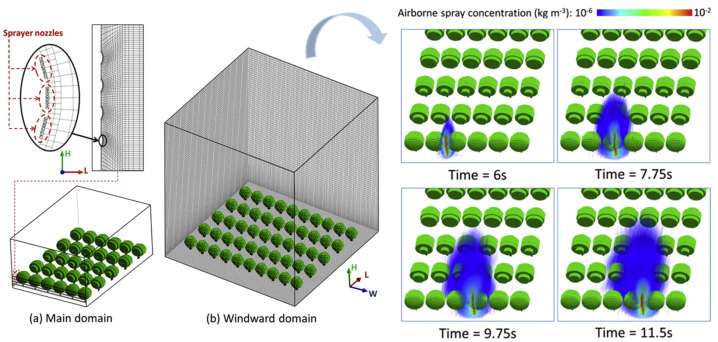
Droplet diffusion and evolution in orchard air-assisted spraying: This figure presents the CFD-simulated three-dimensional diffusion behavior of droplets released by an air-assisted sprayer at 6, 7.75, 9.75, and 11.5 s within an orchard canopy. The simulation focuses on the influence of artificially generated airflow from the sprayer, rather than ambient wind effects. During the initial spraying stage, droplet concentration rapidly attenuates at the front-row canopy interface. As droplets penetrate deeper into the canopy, their dispersion range gradually expands. Turbulence plays a dominant role throughout the process—it disrupts the laminar structure, enhances droplet mixing and canopy penetration, and significantly alters the trajectories and spatial distribution of the droplets. This visualization highlights the dynamic evolution of spray transport under controlled airflow conditions within a heterogeneous canopy environment.

Turbulence further increases the uncertainty of droplet transport pathways. Moderate turbulence is beneficial for disrupting laminar flow structures [75], thereby enhancing droplet mixing and penetration capabilities. However, excessive turbulence intensity may destabilize droplet trajectories, leading to dispersed deposition and reduced targeting accuracy. Turbulence characteristics are typically quantified using turbulent kinetic energy (TKE) and turbulence intensity indices. The response of droplets to turbulent fluctuations is closely related to their size and inertial scale. In simulation studies, computational fluid dynamics (CFD) has been widely employed to analyze droplet trajectories and deposition patterns under varying wind speeds and turbulence conditions. As shown in Figure 7, some studies have adopted numerical modeling approaches with different turbulence models to accurately reproduce the dynamic interaction processes between airflow and spray clouds [76].

However, the orchard wind field itself exhibits significant spatiotemporal uncertainty, driven by a combination of topography, plant arrangement, and meteorological variability. Static wind speed settings are often insufficient to accommodate the dynamically changing wind environment during spraying operations. Therefore, future spray control systems must incorporate enhanced dynamic responsiveness, such as Model Predictive Control (MPC) or time-series forecasting models based on historical wind speed data, to enable feedforward scheduling of spraying strategies.

In summary, wind speed and turbulence structure serve as fundamental variables influencing droplet transport paths and deposition behavior, playing a foundational role in precision spraying systems. The coordinated application of high-fidelity modeling, real-time sensing, and adaptive control strategies holds great promise for improving the robustness and efficacy of spraying operations in complex orchard environments, thereby supporting the practical implementation of intelligent agriculture.

### 3.2. Mechanical Properties and Response Patterns of Leaves

As the primary target surface in orchard spraying systems, plant leaves play a vital role in determining droplet interception, deposition, and adhesion efficiency. Functioning as flexible structures, leaves undergo bending, vibration, and torsion under wind-induced disturbances. These deformations alter the geometric configuration of the target surface and disrupt local airflow, thereby affecting droplet trajectories and deposition stability. Identifying the mechanical response characteristics of leaves is thus essential for optimizing spray timing and control strategies.

The mechanical properties of plant leaves are determined by a combination of geometric dimensions, material characteristics, and structural response parameters [77,78,79], with representative mechanical parameters summarized in Table 2. These properties are influenced by species, developmental stage, water content, and leaf position. Tree species such as apple and pear typically exhibit thicker leaves with higher rigidity and bending resistance, resulting in more stable dynamic responses [80]. In contrast, plants with thinner leaves, such as citrus and grapevine, are prone to large-amplitude oscillations, which cause spray deposition zones to fluctuate over time.

When wind speed approaches a leaf’s natural frequency, resonance may occur, triggering high-amplitude oscillations that act as a “dynamic barrier,” impeding effective droplet deposition and potentially causing rebound or drift. In contrast, leaves with greater structural rigidity maintain more stable orientations, thereby facilitating droplet capture.

**Figure 8:**
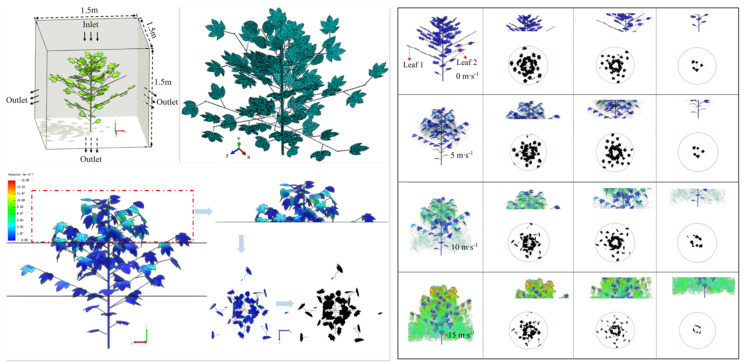
CFD–FSI-based simulation of airflow–structure coupling within the canopy: This figure shows airflow distribution and leaf deformation within a canopy under wind speeds of 0, 5, 10, and 15 m/s using a CFD–FSI approach. The canopy model was reconstructed from three-dimensional leaf point clouds, with simplified petiole structures. Boundary conditions included a top constant-velocity inlet, a zero-pressure outlet, and no-slip walls elsewhere. Airflow was solved using the RNG k–ε model, and leaf flexibility was considered. The **left panel** presents the modeling workflow, including three-dimensional reconstruction and porosity extraction steps; the **right panel** displays airflow fields, deformation patterns, and porosity profiles at different heights. Results indicate that increasing wind speed significantly alters flow penetration and canopy porosity, underscoring the role of aerodynamic–structural coupling in spray dynamics. Reproduced from Cui et al. [81] under the terms of the Creative Commons Attribution License (CC BY 4.0), © Cui, Wang, Lu, Liu, and Yuan (2023), published in Frontiers in Plant Science.

Recent studies have widely adopted fluid–structure interaction (FSI) models based on computational fluid dynamics (CFD) to enable bidirectional simulation of wind–leaf interactions [81], as illustrated in Figure 8. By capturing the feedback effect of leaf deformation on airflow trajectories, a more realistic and dynamically responsive canopy model can be constructed. The simulation examined the variation in canopy porosity under wind speeds ranging from 0 to 15 m/s. Results indicated that at a wind speed of 15 m/s, the porosity in the mid-canopy layer increased by 36.28% compared to that at 5 m/s. This increase was primarily attributed to pronounced leaf bending and structural reconfiguration, which reduced localized aerodynamic resistance and reshaped internal airflow channels.

By integrating the mechanical response characteristics of leaves across different canopy layers with CFD-simulated wind field distributions, this approach provides a theoretical basis for determining layer-specific wind speed and airflow volume settings in variable-rate spraying systems.

In addition, some studies have employed high-speed imaging and image processing techniques to capture leaf displacement, oscillation frequency, and deformation modes under wind exposure, providing high-resolution data for model validation [53]. In practical spray control applications, recognizing the dynamic state of leaves can help optimize spray timing. Initiating spraying during the stabilization phase of leaf oscillation has been shown to improve hit rate and deposition uniformity. Certain intelligent spraying systems have already integrated visual recognition modules for leaf motion monitoring, combining depth cameras with image analysis algorithms to establish a closed-loop control mechanism characterized by “target detection—dynamic response—precision spraying.”

However, several challenges remain in this area. First, variability in plant species and growth stages limits the generalizability of mechanical models. Second, the inherent instability of natural wind fields introduces randomness in leaf responses, increasing model uncertainty. Third, research on the coordinated response of multiple leaves is still limited, and the collective regulation mechanisms of leaf clusters on local droplet fields remain poorly understood.

In summary, leaf mechanical responses not only influence their physical state as spray targets, but also indirectly modulate droplet deposition trajectories by perturbing the local airflow. Future work should emphasize the integration of leaf structural modeling with real-time perception systems, advancing toward adaptive spray control strategies based on dynamic response features. Such developments will enhance the intelligence and robustness of orchard spraying systems.

### 3.3. Physical Properties of Droplets and Deposition Behavior

As the fundamental carriers of pesticides in spraying systems, droplet physical properties play a decisive role in determining transport efficiency, target-specific deposition, and environmental safety. Key influencing factors include droplet size [82], initial velocity, electrical charge state [83], surface tension, liquid viscosity, and volatility [84,85,86]. These parameters jointly affect the aerodynamic stability of droplets, their adhesion behavior upon leaf contact, and the overall deposition outcome.

Droplet size is one of the most critical dynamic parameters influencing spray performance. Large droplets possess high inertia, making them suitable for deposition on the outer canopy surface or tree trunks, but they exhibit poor penetration capability. In contrast, fine droplets are more easily carried by airflow into inner canopy layers, making them ideal for deep-targeted spraying, though they are more susceptible to drift.

**Figure 9:**
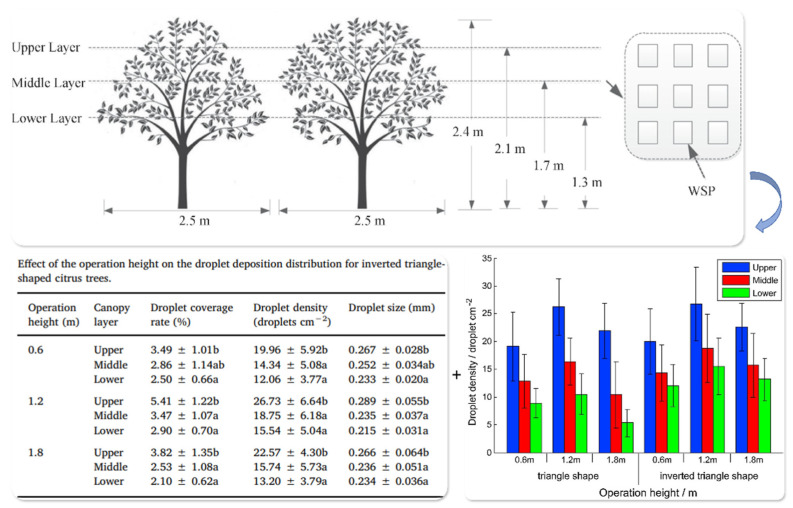
Relationship between droplet size, deposition efficiency, and penetration performance across canopy layers at different spraying heights: This figure illustrates the response patterns of droplet deposition characteristics to droplet size variation within the upper, middle, and lower canopy layers of an inverted-cone-shaped citrus tree. Measurements were conducted at three operating heights (0.6 m, 1.2 m, and 1.8 m), recording droplet coverage rate, deposition density, and volume median diameter (VMD) for each layer. The results show a gradual decrease in droplet size from the upper to the lower canopy layers. Notably, at the 1.2 m spraying height, the deposition efficiency in the middle and lower layers was significantly improved, indicating that smaller droplets have stronger penetration capacity into the canopy interior. In contrast, larger droplets were primarily distributed on upper leaves, demonstrating a size-dependent stratified deposition behavior. Different lowercase letters (e.g., a, b) following the values indicate statistically significant differences (*p* < 0.05) among canopy layers at the same spraying height based on Duncan’s multiple range test.

Studies have shown that differentiated droplet size strategies should be applied to match specific target zones within the canopy, balancing deposition efficiency and penetration performance. This enables stratified and directional application suitable for diverse orchard spraying scenarios [87]. As shown in Figure 9, field measurements under UAV-based spraying conditions reveal a strong correspondence between droplet size distribution and deposition responses across different canopy layers at varying flight altitudes. These findings highlight the potential of UAV systems in regulating both penetration and deposition uniformity. Compared with traditional ground-based sprayers, UAVs offer superior altitude flexibility and adaptability to target zones, presenting a promising engineering pathway for achieving stratified and site-specific spraying.

Droplet velocity influences both inertial force and impact energy, thereby affecting wind resistance and deposition probability [88]. Higher spray velocities enhance penetration and adhesion strength, but may also lead to droplet breakup [89], rebound, or even “overshooting” the target, particularly when leaves are undergoing significant oscillation. Therefore, optimal spray velocity should be adapted to factors such as leaf stiffness and ambient wind conditions.

**Figure 10:**
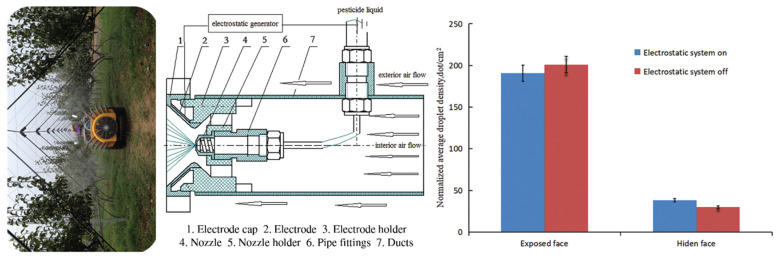
Configuration of a dual air-assisted electrostatic spraying system and its deposition performance on different leaf surfaces: The left panel shows the field operation of a dual-fan electrostatic orchard sprayer in a pear orchard. The middle panel illustrates the structure and working principle of the electrostatic nozzle. The right panel compares the normalized droplet deposition density on the exposed and hidden (abaxial) leaf surfaces with and without electrostatic charging. The results indicate that activation of the electrostatic system increased droplet deposition on the hidden leaf surface by 29.4%, while deposition on the exposed surface slightly decreased by 5.4%. The enhanced electrostatic attraction improved adhesion to concealed targets such as the underside of leaves, thereby significantly improving deposition uniformity in otherwise hard-to-reach canopy areas.

Electrostatic spraying enhances droplet adhesion through electrical charging, increasing deposition efficiency under the influence of electrostatic fields around leaves [90,91,92]. This approach is particularly effective for reaching concealed areas such as the undersides of leaves. Charged droplets exhibit a “reverse adhesion” phenomenon under positive–negative polarity induction, with studies reporting up to a 30% increase in deposition efficiency [93,94], as illustrated in Figure 10. However, the effectiveness of electrostatic spraying is influenced by multiple interacting variables: higher voltages increase charge density but pose risks of corona discharge; smaller droplets acquire charge more readily but are more prone to environmental interference. Thus, the system must be holistically optimized across voltage level, droplet size, velocity, and ambient humidity.

In modeling and simulation, Lagrangian particle tracking combined with the Volume of Fluid (VOF) method have been employed to predict droplet transport trajectories and deposition distribution patterns. Multiphase flow models further incorporate droplet evaporation dynamics to evaluate pesticide loss under high-temperature conditions. When applied to three-dimensional orchard canopy structures, these modeling approaches enable thermal sensitivity analysis of droplet deposition across different canopy layers. This provides valuable guidance for optimizing droplet size, airflow velocity, and spray volume settings, particularly in the middle and lower canopy regions. By simulating spray transport efficiency under complex climatic and structural conditions, such models offer theoretical support for multilayer spraying strategies and parameter configuration in variable-rate control systems.

In summary, droplet size, velocity, electrical charge state, and physicochemical properties of the spray liquid are key variables determining droplet deposition behavior in complex orchard environments. By establishing a multi-parameter coordinated control strategy and incorporating environmental sensing with canopy-structure-aware zonal management, it is possible to significantly enhance targeted deposition efficiency, reduce drift risk, and achieve high-efficiency, precision-oriented, and environmentally sustainable spraying operations.

### 3.4. Canopy Structural Parameters and Penetration Characteristics

As the primary medium for droplet transport and deposition in spraying systems, the structural characteristics of the plant canopy play a decisive role in determining droplet penetration pathways, interception efficiency, and final deposition distribution. The canopy structure is composed of multiple interrelated factors, including leaf area index (LAI) [95,96], leaf density, layer thickness, spatial arrangement of branches, porosity, and both vertical and horizontal heterogeneity. These features collectively define the canopy as a typical multiscale, heterogeneous porous medium, exhibiting strong filtration capacity and directional guidance for droplet flow.

**Figure 11:**
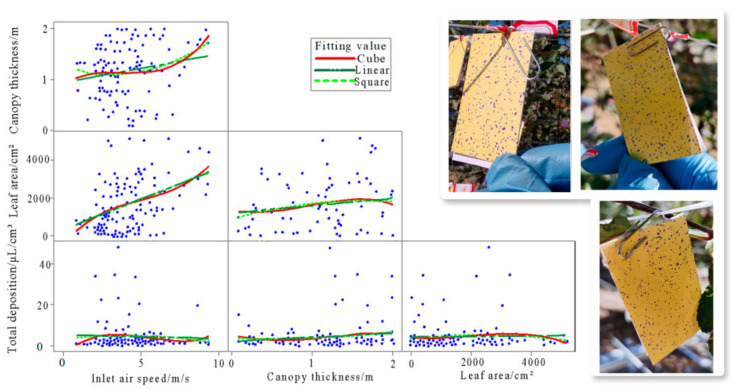
Visual analysis of the effects of leaf area and canopy structure on droplet deposition distribution: The left panel illustrates the effects of wind speed, canopy thickness, and leaf area on droplet deposition, while the right panel presents the corresponding water-sensitive paper sampling results. Fitting model analysis reveals that with increasing canopy thickness and leaf area, droplet deposition exhibits non-linear fluctuations, characterized by a distinct gradient pattern—higher deposition in the outer layers and reduced deposition in the inner canopy, particularly in high leaf area regions. Among the tested models, the quadratic fit yielded the best performance, with coefficients of determination (R^2^) of 0.018 for wind speed, 0.014 for canopy thickness, and 0.016 for leaf area, indicating moderate explanatory power for these structural variables in influencing spray distribution.

Among these factors, leaf area index (LAI) is a key indicator for evaluating canopy density. A higher LAI value typically corresponds to a denser outer canopy with greater interception efficiency; however, it also impedes droplet penetration into the inner canopy, resulting in non-uniform deposition characterized by surface overspray and interior underspray. As shown in Figure 11, research has demonstrated that larger total leaf area leads to more densely packed foliage within a given volume, thereby increasing the interception of spray droplets by outer leaves. This causes a higher proportion of the pesticide to deposit on the canopy surface, with limited penetration into the inner layers [97]. Specifically, in regions with a leaf area greater than 3000 cm^2^, the deposition on inner water-sensitive papers was significantly lower than that on the outer ones, indicating a clear imbalance. Therefore, in dense canopies, strategies such as increasing droplet kinetic energy or employing penetration-enhancing techniques are recommended to improve inner canopy coverage.

**Figure 12:**
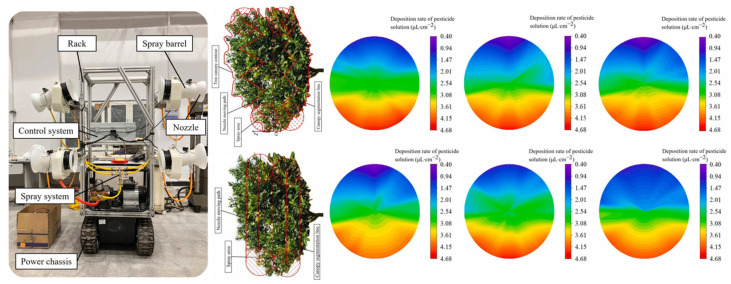
Vertical canopy structure and workflow of a variable-rate spraying control system in fruit orchards: Workflow of a LiDAR-based variable-rate spraying control system for fruit tree canopies. The system performs real-time scanning to acquire canopy point cloud data, followed by volume estimation, boundary recognition, and trajectory matching to enable precise, stratified pesticide application across different vertical canopy layers (upper, middle, lower). A boom pitch-angle control strategy is employed to effectively address the common issue of “saturated upper-layer deposition—insufficient lower-layer deposition,” thereby enhancing droplet delivery to the lower canopy regions.

The vertical stratification of the canopy is another critical factor influencing the distribution of spray deposition across different layers. Upper leaves intercept droplets first and consume their kinetic energy, limiting the deposition potential on lower layers. In multilayer canopy structures—such as those found in apple and pear trees—a typical pattern of “saturated deposition on the upper layer—weak deposition on the lower layer” is often observed. To address this issue, some studies have explored biomimetic multi-angle nozzle designs [98], which enhance droplet delivery to the lower canopy regions, as illustrated in Figure 12.

In recent years, the integration of LiDAR (Light Detection and Ranging) [99] and multispectral sensors [100] has supported the accurate acquisition of canopy parameters. LiDAR enables the construction of dense three-dimensional point clouds from which spatial features such as LAI, porosity, and leaf distribution can be extracted, providing a solid basis for site-specific and stratified spray management. Future research will increasingly focus on the use of deep learning algorithms for intelligent interpretation of point cloud data, enabling real-time reconstruction of canopy structural maps to support data-driven variable-rate spraying strategies.

CFD simulations have further elucidated the regulatory mechanisms by which canopy structure influences airflow patterns [101] and droplet behavior [102,103], enabling the modeling of wind speed attenuation, vortex distribution, and droplet deposition trajectories. However, canopy structures exhibit pronounced spatiotemporal dynamics, continuously evolving under the influence of seasonal variation, pruning operations, and tree growth status. These changes lead to ongoing fluctuations in structural parameters. As a result, spraying systems must be equipped with dynamic sensing capabilities to capture real-time canopy data and adapt spray strategies accordingly, thereby establishing a closed-loop optimization framework that links structure, response, and control.

In summary, canopy structure plays a dual role in spraying systems—as both a filtration barrier and a guiding medium—with its parameters directly affecting spray uniformity and targeting precision. Future intelligent spraying systems should aim to improve canopy modeling accuracy, incorporate real-time structural sensing, and implement region-specific variable control mechanisms. These advancements will enable the realization of a “structure-driven, sensing-enabled, and strategy-responsive” precision application paradigm.

## 4. Modeling and Experimental Advances

### 4.1. Multiscale Modeling Approaches: CFD, FSI, and Data-Driven Methods

The wind–leaf–droplet system involves a complex interplay of physical phenomena, including fluid dynamics, structural mechanics, and particle kinematics. Its modeling requires careful consideration of multiscale coupling and dynamic response behaviors. Currently, multiscale modeling frameworks are primarily built upon computational fluid dynamics (CFD), and are increasingly integrated with fluid–structure interaction (FSI) models and data-driven approaches, resulting in hybrid modeling systems with enhanced structural completeness and adaptability.

**Figure 13:**
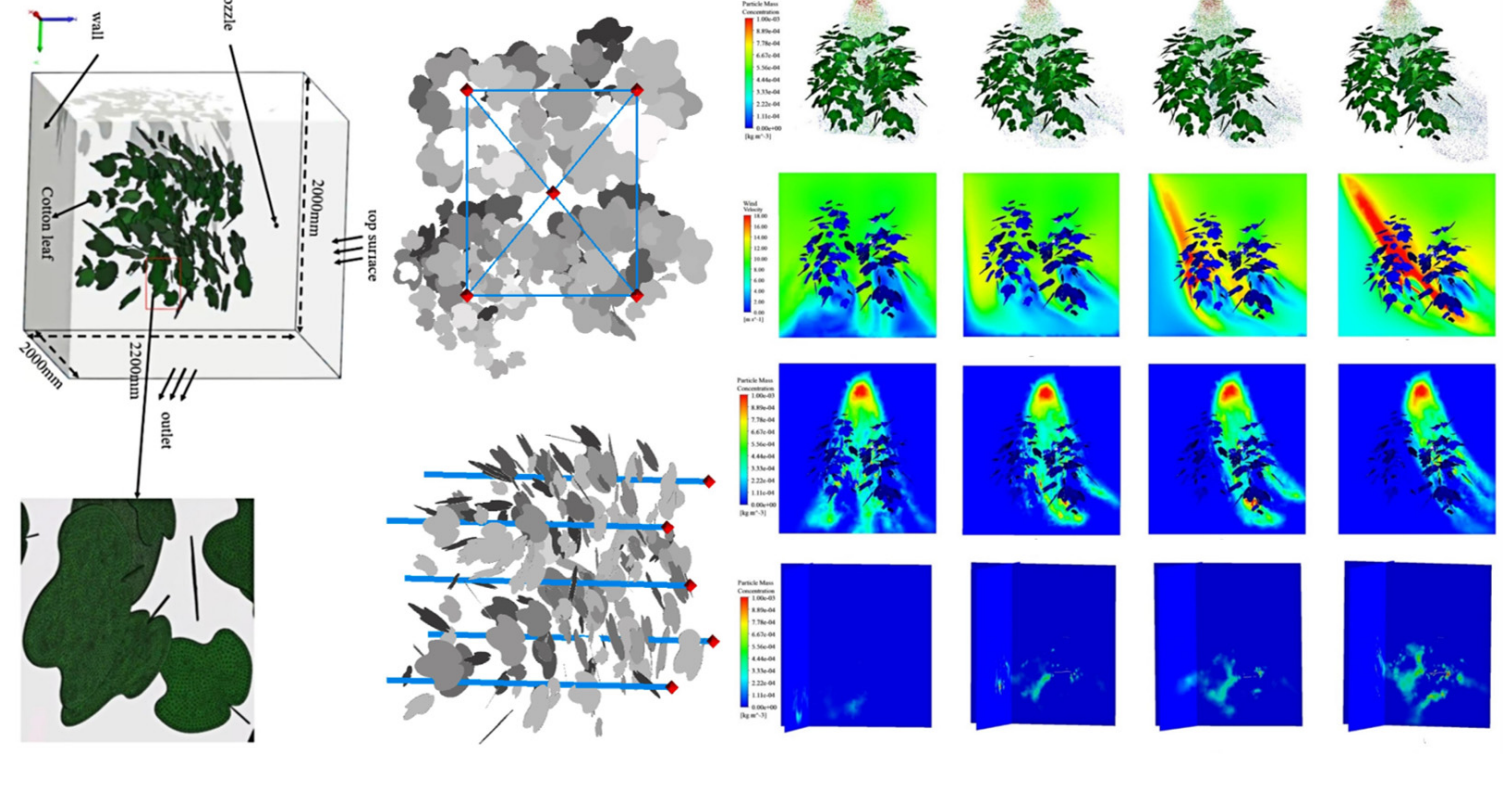
CFD simulation of droplet deposition and drift behavior under varying natural wind speeds: This figure presents CFD-based simulations of droplet particle trajectories, deposition patterns, and drift behavior within the canopy under four natural wind speeds (0, 1, 3, and 5 m/s) in an air-assisted spraying system. The sprayer emits a vertical downward airflow at 10 m/s with a spray rate of 0.28 L/min, while a crosswind blows west to east, orthogonal to the main airflow. The simulations reveal that as wind speed increases, droplet trajectories deviate more significantly before canopy entry, with some particles diverted from the central path due to diffraction, deflection, or lateral drift. Airflow disturbances also reconstruct the internal canopy wind field, leading to stratified deposition patterns and evident off-target deposition in outer zones. Wind speed has a pronounced impact on particle transport pathways, penetration depth, and deposition uniformity.

CFD remains the core methodology for simulating wind–droplet interactions. By solving the Navier–Stokes equations and incorporating appropriate turbulence models, CFD can accurately simulate wind speed attenuation within the canopy [102], recirculation zones, and turbulence structures. When combined with Lagrangian particle tracking and multiphase flow modeling [104], CFD enables the prediction of droplet transport trajectories, drift behavior, and deposition patterns, as illustrated in Figure 13. For higher-resolution modeling, some studies have adopted Volume of Fluid (VOF) or gas–liquid two-phase models [105,106,107] to reconstruct the dynamic processes of droplet fragmentation, coalescence, and evaporation at the group level.

However, traditional CFD approaches exhibit certain limitations when simulating the dynamic response of flexible plant leaves, as they often treat leaves as rigid, static bodies. This simplification fails to capture the actual deformation behavior of leaves under wind loading and their feedback effects on local airflow and droplet transport. To address this, fluid–structure interaction (FSI) models have been introduced, wherein the CFD fluid domain is coupled with a finite element-based structural domain via Arbitrary Lagrangian–Eulerian (ALE) or domain partitioning techniques. This enables bidirectional dynamic interaction between wind fields and leaf motion. FSI significantly improves the accuracy of wind–leaf–droplet interaction modeling and facilitates the identification of optimal spray windows and high-risk drift zones, providing a physically grounded foundation for responsive spraying strategies. Nonetheless, such models still face challenges including high computational costs, complex parameterization, and difficulties in maintaining cross-scale coupling consistency. Their applicability and generalizability under field conditions require further validation.

**Figure 14:**
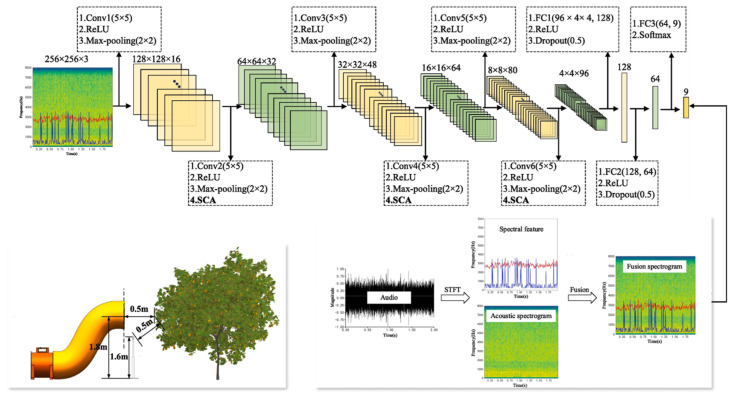
Model architecture and feature extraction pipeline for canopy leaf density prediction based on wind-induced audio signals: The figure illustrates the complete modeling workflow for predicting fruit tree canopy leaf density using wind-induced acoustic signals. The **lower left** panel shows the spatial configuration of the wind source relative to the canopy. The **lower right** panel depicts the transformation of raw audio signals through Short-Time Fourier Transform (STFT) to extract spectral features and generate a spectrogram, which is then fused into an enhanced Frequency Spectrum Plot (FSP). The **upper panel** presents the structure of the deep convolutional neural network (SCA-DCNN), which takes the FSP as input and incorporates a Spectral Centroid Attention (SCA) mechanism to enhance sensitivity to key frequency-domain features, thereby improving the accuracy of leaf density prediction.

With the development of agricultural sensing and big data technologies, data-driven modeling methods have emerged as a powerful complement. By integrating multi-source empirical data—such as wind speed, leaf state, and deposition maps—with deep learning and machine learning models [108,109,110], researchers have established input–output mappings that can reveal complex multiscale interactions during spraying, including droplet drift and fragmentation [111], leaf deformation, and localized airflow disturbances [112]. These models enable rapid prediction and regional control of spray effectiveness. For instance, one study extracted frequency-domain features from audio signals generated by wind–leaf interactions and developed a deep convolutional neural network (SCA-DCNN) that integrates a Frequency Spectrum Plot (FSP) and a Spectral Centroid Attention (SCA) mechanism [113], as illustrated in Figure 14. This model successfully mapped wind-induced canopy responses to leaf density, offering a novel pathway for multiscale dynamic modeling and predictive analysis in precision spraying applications.

Current research trends are increasingly moving toward the integration of physics-based modeling and data-driven approaches, leading to the development of either physics-constrained data models or data-enhanced numerical models. The former leverages physical boundaries and laws as priors to improve model generalizability, while the latter uses empirical data to compensate for the simplifications inherent in traditional physical models, thereby enhancing predictive efficiency. This hybrid modeling paradigm offers a promising pathway to balance accuracy and computational efficiency, providing more robust and scalable solutions for intelligent orchard spraying systems.

### 4.2. Experimental Observation and Visualization Technologies

Due to the highly dynamic and multiscale coupled nature of the wind–leaf–droplet system, it remains difficult to analyze its internal mechanisms through modeling alone. Therefore, experimental observation and visualization technologies serve as essential tools for elucidating physical interactions, validating simulation results, and informing control strategy optimization. In recent years, rapid advances in optical imaging, laser-based measurement, and three-dimensional reconstruction technologies have greatly enhanced system-level understanding of wind–leaf–droplet interactions.

In wind field observation, Particle Image Velocimetry (PIV) is among the most widely used techniques [114]. PIV involves seeding tracer particles into the flow, illuminating them with laser sheets, and capturing high-speed images to derive two- or three-dimensional velocity vector fields in a non-invasive manner. This method is commonly applied in wind tunnel experiments and localized ventilation analysis [115], enabling precise identification of wind speed attenuation zones, vortex structures, and flow reconstruction patterns. These outputs provide boundary condition support for flow field modeling and fluid–structure interaction simulations.

**Figure 15:**
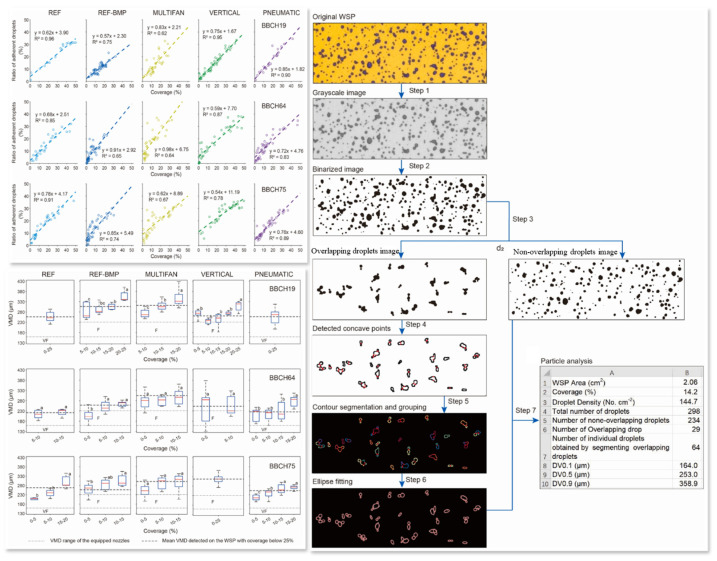
Spray deposition pattern analysis on water-sensitive paper using overlapped droplet segmentation: This figure illustrates an image segmentation method designed for analyzing overlapping droplets, including steps such as grayscale conversion, binarization, circularity filtering, concave point detection, contour segmentation, and elliptical fitting. This approach enables precise extraction of droplet count and morphological parameters. Based on this method, key spray deposition metrics can be quantified, including droplet size distribution (DV0.1, DV0.5, DV0.9), coverage rate, and the proportion of overlapping droplets. The **left panel** presents the fitted relationships between droplet density and coverage rate under three growth stages (BBCH19, BBCH64, BBCH75) and five sprayer configurations. Results show a significant positive correlation between the two variables under low coverage conditions (<25%), with R^2^ values exceeding 0.7. This finding supports the establishment of quantitative links between droplet image features and overall deposition distribution.

For droplet behavior analysis and quantification, water-sensitive paper and chemical extraction methods are commonly employed. The former is simple, low-cost, and well-suited for rapid field assessment in orchards [116,117], as shown in Figure 15, though it is limited in spatial resolution and quantitative precision. The latter uses spectrophotometric analysis of dye concentrations to accurately determine deposition levels, making it suitable for dose validation, albeit lacking real-time capability. Laser-Induced Fluorescence (LIF) [118] offers high sensitivity, non-contact measurement, and dynamic tracking, and has become a valuable tool for studying multilayer canopy penetration and deposition distributions. However, its high cost and limited environmental adaptability currently constrain its broader application in open-field conditions.

**Figure 16:**
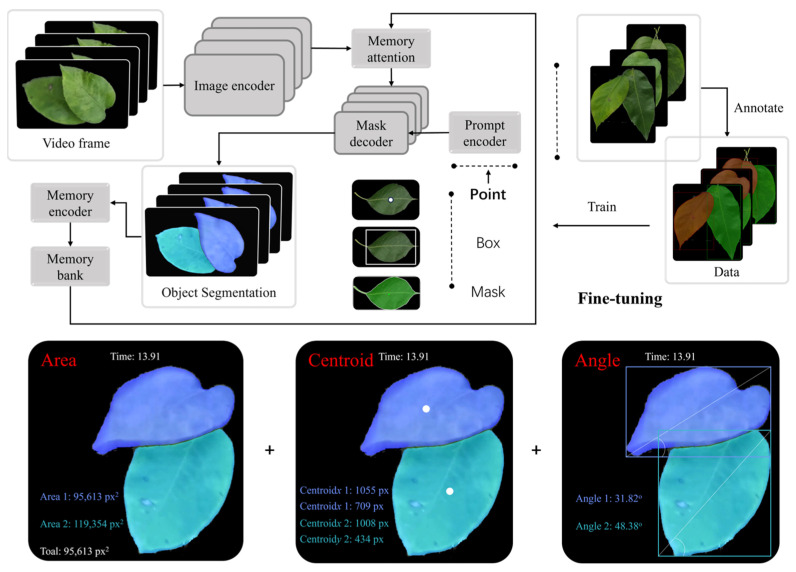
Analysis of leaf dynamic behavior under wind-driven conditions: This figure illustrates the complete workflow for temporal segmentation, tracking, and dynamic behavior extraction of wind-induced leaf motion using the fine-tuned SAM2-FT model. The **upper section** presents the model architecture and inference pipeline, including image encoding, memory attention mechanism, and mask decoding modules. The **middle section** displays pear leaf video data used for training and testing, along with initial annotations in the form of points, bounding boxes, or masks. The **lower section** shows the extraction results of three key dynamic variables: leaf area (Area), representing morphological changes; centroid (Centroid), indicating motion trajectory; and tilt angle (Angle), reflecting posture response. These metrics provide a dynamic data foundation for modeling wind–leaf interaction and optimizing spray strategies.

In the observation of leaf dynamic responses, machine vision combined with deep learning techniques have been employed to reconstruct leaf geometry and motion trajectories [119], as illustrated in Figure 16. By integrating image processing algorithms, it is possible to extract vibration frequency, swing amplitude, and modal shape characteristics of leaves. These features provide a solid foundation for constructing structural models and identifying optimal spray response windows.

High-speed imaging technology (>1000 fps) is primarily applied at the microscale level [120,121,122] to capture the interactions between droplets and leaf surfaces during impact events, including spreading [123], rebound, and coalescence behaviors. When combined with backlit imaging and fluorescent labeling, high-speed cameras can record detailed droplet deformation sequences, providing empirical support for the validation of wetting and adhesion models. Some studies have also integrated infrared thermography to monitor leaf surface temperature changes induced by spraying, enabling indirect estimation of evaporation rates and deposition uniformity—particularly useful in electrostatic spraying or thermosensitive formulation scenarios.

Despite continual improvements in measurement precision, field-based experimentation still faces notable challenges such as uneven lighting, severe occlusion, and limited observation angles. Future research should focus on advancing toward portable, automated, and real-time solutions. For example, deploying UAV-mounted sensors for in-field canopy structure measurement, or developing embedded systems for networked data acquisition during spraying operations, would significantly enhance the intelligent sensing and responsive control capabilities of orchard spraying systems.

## 5. Challenges and Future Research Directions

### 5.1. Current Research Challenges

Although multiscale modeling of wind–leaf–droplet interactions plays a vital role in revealing spray dynamics and optimizing pesticide application strategies, significant challenges remain in terms of model construction, parameter acquisition, and validation techniques.

First, limited coupling across subsystems. Most current studies address individual components—such as wind field perturbation (CFD), flexible leaf response (FSI), or droplet transport (particle dynamics, wetting models)—in isolation, lacking an integrated, cross-scale framework capable of dynamically describing the interactions among wind, droplets, and leaves. Subsystems vary significantly in spatial scale, temporal resolution, and response mechanisms, leading to compatibility issues in boundary condition handling, system instability, and limited real-time applicability, which severely constrain the scalability and adaptability of these models in field applications.

Second, difficulty in parameter acquisition. Critical model inputs—such as natural wind characteristics, three-dimensional canopy architecture, leaf mechanical properties, and droplet size distribution—are strongly influenced by orchard-specific factors including terrain, species, and management practices, making rapid and standardized acquisition challenging. As a result, model inputs often contain significant uncertainty, which compromises simulation accuracy and generalizability.

Third, limited validation dimensionality. While high-speed photography has been used to capture high-resolution spatiotemporal sequences of droplet–leaf interactions (e.g., impact, spreading, rebound, and shatter), automated recognition and structured analysis of these processes remain underdeveloped due to data complexity and volume. Moreover, as leaves behave as flexible structures during spraying, their wind-induced responses—such as vibration frequency, oscillation amplitude, postural changes, and morphological reconfiguration—can significantly modulate local airflow patterns and droplet trajectories. Yet, most existing studies focus on single-leaf displacement extraction or qualitative observations, lacking refined modeling of collective leaf dynamics and feedback mechanisms.

Currently, experimental validation efforts still rely heavily on indirect indicators such as deposition rate and surface coverage, lacking high-resolution visualization and quantitative feedback on localized droplet–leaf interactions. This limitation hinders the calibration of causal mechanisms and the identification of responsive windows during the modeling process.

Existing modeling approaches vary in their reliability when aligned with empirical data. Static models are suitable for approximating general canopy structures but often fail to capture spatiotemporal consistency. Quasi-dynamic models can reflect certain leaf responses but struggle to address the non-linear characteristics of interactions between actual wind fields and droplet transport. In contrast, dynamic models—such as CFD coupled with FSI—offer superior fidelity, particularly in simulating leaf deformation, droplet distribution, and airflow interaction. However, these methods entail high data demands and computational complexity. Future work should integrate deep learning and intelligent image analysis techniques to develop video-level behavior recognition and evaluation models that capture the bidirectional interactions between droplets and leaves. Such models would enable coupled feedback modeling of structural responses and droplet transport, promoting a closed-loop integration between multiscale modeling and experimental observation.

### 5.2. Future Research Directions

To overcome the above limitations and enhance the modeling, sensing, and control capabilities of wind–leaf–droplet systems, future research should focus on the following four directions to advance both theoretical methods and field applications:

1. Develop unified cross-scale coupling platforms. Integrate CFD (wind field), FSI (structural response), and droplet transport/wetting models into a chain-based modeling framework, capturing the entire process from external wind disturbance to leaf dynamics and eventual droplet deposition. This enables continuous multiscale representation and response window identification.
2. Establish intelligent observation systems with experimental feedback. Combine high-speed imaging, machine vision, and semantic image recognition techniques with deep learning to structurally decode droplet–leaf interactions—including droplet deformation, contact behavior, spreading, and adhesion dynamics—providing fine-grained calibration data to transition from image recording to mechanistic interpretation.
3. Promote integration of physics-based and data-driven models. Develop physics-informed machine learning (PIML) frameworks that embed governing equations, causal logic, and observational data into the training process. This enhances model interpretability, robustness, and adaptability across environmental conditions, addressing the challenges of sparse data, complex physical behavior, and model instability.
4. Construct closed-loop systems of sensing, modeling, and control. Integrate multimodal sensors (e.g., RGB, infrared, multispectral, LiDAR point clouds) into orchard operation platforms to support real-time environmental perception for spraying tasks. By incorporating edge computing and reinforcement learning, these systems can achieve intelligent, adaptive control for variable-rate spraying.

Through the coordinated development of these four directions, it is anticipated that the field will progress toward closed-loop optimization across the entire pipeline—from fundamental mechanistic understanding to intelligent operational control—thereby driving orchard spraying technologies toward higher efficiency, adaptability, and intelligence.

## 6. Conclusions

The multiscale coupling mechanisms of the wind–leaf–droplet system represent a fundamental process that governs spray efficiency and pesticide utilization in orchard environments. This review has systematically examined the regulatory effects of microscale droplet wetting behavior, mesoscale coordinated leaf responses, and macroscale canopy structural characteristics on wind–droplet flow reconstruction, highlighting the complex dynamic interactions across spatial scales.

On the theoretical side, computational fluid dynamics (CFD), fluid–structure interaction (FSI), and data-driven approaches have begun to form an integrated modeling framework for wind–leaf–droplet interactions. Experimentally, the application of deep learning, three-dimensional vision, and high-resolution observation techniques has significantly enhanced system-level monitoring and model validation capabilities. Furthermore, the review summarized the functional pathways of key variables in spraying systems, laying a theoretical foundation for deeper insights into spray dynamics and the development of optimized application strategies.

It is worth noting that our research team has conducted a series of relevant studies in this domain. These include investigating the droplet breakup mechanisms of a self-developed nozzle using a high-speed imaging system; developing a leaf tracking and detection model to quantify wind–leaf interaction dynamics; and integrating LiDAR and visual sensors for orchard environment perception and spray control. These practical achievements provide strong support for the research framework and future directions proposed in this review. Moreover, they validate the feasibility and application potential of key modeling pathways in real-world scenarios.

Beyond summarizing current progress, this work has explored future directions for cross-scale model integration, hybrid data–physics modeling, and standardized experimental validation. By building unified modeling platforms and advancing highly adaptive sensing and control technologies, more intelligent and efficient solutions can be envisioned for sustainable pest and disease management in orchards. Overall, the study of wind–leaf–droplet multiscale interactions is now at a pivotal stage of transitioning from theoretical investigation to engineering application.

## Figures and Tables

**Table 1:** Effects of surfactants and adjuvants on droplet–leaf surface interactions.

Researcher	Plant Type	Main Methodology	Key Findings	Ref.
Zhou et al.	Cotton leaves and droplets	Measurement of droplet volume, height, and contact angle evolution	Reduced droplet slippage and improved pesticide retention	[42]
Liu et al.	Citrus leaves and droplets	Contact angle and surface tension analysis	Significantly reduced surface tension and contact angle; enhanced wettability	[43]
Gao et al.	Tea leaves and droplets	Contact angle measurement and SEM (Scanning Electron Microscopy) analysis	Lowered contact angle and accelerated droplet spreading	[44]
Arand et al.	Weed leaves and droplets	Evaluation of droplet retention, hydration, and cuticle penetration	Improved efficacy by enhancing droplet retention and penetration	[45]
Meng et al.	Wheat leaves and droplets	Dynamic contact angle and wetting velocity testing	Reduced contact angle and increased wetting efficiency	[46]

**Table 2:** Classification and functional roles of key parameters influencing leaf mechanical properties.

Category	Representative Parameter	Physical Significance/Role	Potential Influence
Geometric Parameters	Leaf length	Determines overall flexibility and inertial response	Longer leaves tend to have lower stiffness and are more prone to large deformations
	Leaf thickness	Affects bending stiffness and total mass	Greater thickness increases stiffness, resulting in slower dynamic response
	Petiole length	Governs flexibility of the connecting segment	Longer petioles may reduce system damping
Material Properties	Young’s modulus	Indicates material rigidity	Higher modulus corresponds to greater stiffness and smaller deformation
	Density	Determines leaf mass and inertial characteristics	Higher density may lower the natural response frequency
Dynamic Response Parameters	Natural frequency	Dominant frequency under free vibration	Jointly influenced by mass and stiffness
	Damping coefficient	Represents the system’s ability to dissipate vibrational energy	Higher damping leads to faster stabilization of the system

## Data Availability

No new data were created or analyzed in this study. Data sharing is not applicable to this article.

## References

[1] Wang A., Li W., Men X., Gao B., Xu Y., Wei X. (2022). Vegetation detection based on spectral information and development of a low-cost vegetation sensor for selective spraying. Pest Manag. Sci..

[2] Zhang S., Qiu B., Xue X., Sun T., Peng B. (2020). Parameters optimization of crop protection UAS based on the first industry standard of China. Int. J. Agric. Biol. Eng..

[3] Wan L., Li H., Li C., Wang A., Yang Y., Wang P. (2022). Hyperspectral Sensing of Plant Diseases: Principle and Methods. Agronomy.

[4] Zhu H., Wang D., Wei Y., Zhang X., Li L. (2024). Combining Transfer Learning and Ensemble Algorithms for Improved Citrus Leaf Disease Classification. Agriculture.

[5] Poulsen M.E., Wenneker M., Withagen J., Christensen H.B. (2012). Pesticide residues in individual versus composite samples of apples after fine or coarse spray quality application. Crop Prot..

[6] Mahmud M.S., Zahid A., He L., Martin P. (2021). Opportunities and Possibilities of Developing an Advanced Precision Spraying System for Tree Fruits. Sensor.

[7] Zhou Q., Xue X., Chen C., Cai C., Jiao Y. (2023). Canopy deposition characteristics of different orchard pesticide dose models. Int. J. Agric. Biol. Eng..

[8] Xun L., Campos J., Salas B., Fabregas F.X., Zhu H., Gil E. (2023). Advanced spraying systems to improve pesticide saving and reduce spray drift for apple orchards. Precis. Agric..

[9] Yu S., Cui L., Cui H., Liu X., Liu J., Xin Z., Wang D. (2024). Spray performance of flexible shield canopy opener and rotor wind integrated boom-sprayer application in soybean: Effects on droplet deposition distribution. Pest Manag. Sci..

[10] Wu S., Liu J., Wang J., Hao D., Wang R. (2021). The motion of strawberry leaves in an air-assisted spray field and its influence on droplet deposition. Trans. ASABE.

[11] Gao J., Tunio M.H., Chen Y., He R. (2020). Design and experiment of low-frequency ultrasonic nozzle integrating air-assistant system and acoustic levitation mechanism. Int. J. Agric. Biol. Eng..

[12] Gao J., Xu K., He R., Chen X., Tunio M.H. (2022). Development and experiments of low frequency ultrasonic electrostatic atomizing nozzle with double resonators. Int. J. Agric. Biol. Eng..

[13] Hu W., Gao Z., Dong X., Chen J., Qiu B. (2024). Contact Electrification of Liquid Droplets Impacting Living Plant Leaves. Agronomy.

[14] Guo J., Dong X., Qiu B. (2024). Analysis of the Factors Affecting the Deposition Coverage of Air-Assisted Electrostatic Spray on Tomato Leaves. Agronomy.

[15] Xi T., Li C., Qiu W., Wang H., Lv X., Han C., Ahmad F. (2020). Droplet deposition behavior on a pear leaf surface under wind-induced vibration. Appl. Eng. Agric..

[16] Liu Z., Chen J., Guo J., Qiu B. (2024). Numerical Simulation and Validation of Droplet Deposition on Tomato Leaf Surface under Air-Assisted Spraying. Agronomy.

[17] Wang Q., Ren Y., Wang H., Wang J., Yang Y., Zhang Q., Zhou G. (2024). Wind-induced response of rapeseed seedling stage and lodging prediction based on UAV imagery and machine learning methods. Comput. Electron. Agric..

[18] Cao Y., Wang C., An Y., Chen Y., Qiu W. (2024). Droplet deposition behavior on the surface of wheat leaves with wind-induced vibration. Crop Prot..

[19] Gong C., Li D., Kang C. (2022). Visualization of the evolution of bubbles in the spray sheet discharged from the air-induction nozzle. Pest Manag. Sci..

[20] Ma J., Liu K., Dong X., Chen C., Qiu B., Zhang S. (2022). Effects of Leaf Surface Roughness and Contact Angle on In Vivo Measurement of Droplet Retention. Agronomy.

[21] Wang H., Shi H., Li Y., Wang Y. (2014). The effects of leaf roughness, surface free energy and work of adhesion on leaf water drop adhesion. PLoS ONE.

[22] Jiang H., Xin D., Zhang H. (2021). Wind-tunnel study of the aerodynamic characteristics and mechanical response of the leaves of betula platyphylla sukaczev. Biosyst. Eng..

[23] Li J., Shi Y., Lan Y., Guo S. (2019). Vertical distribution and vortex structure of rotor wind field under the influence of rice canopy. Comput. Electron. Agric..

[24] Liu J., Liu X., Zhu X., Yuan S. (2016). Droplet characterisation of a complete fluidic sprinkler with different nozzle dimensions. Biosyst. Eng..

[25] Xia H., Zhang X., Xiao J. (2022). Breakup behavior of a shear-thinning droplet on randomly rough surfaces: A numerical study. Chem. Eng. Sci..

[26] Yue J., Chao C., Hong L., Qingjiang X. (2016). Influences of nozzle parameters and low-pressure on jet breakup and droplet characteristics. Int. J. Agric. Biol. Eng..

[27] Pan X., Jiang Y., Li H., Hui X., Xing S., Chauhdary J.N. (2024). Numerical simulation and experimental study of jet breakup using a water dispersal needle in irrigation sprinklers. Biosyst. Eng..

[28] Jiang Y., Yang Z., Xu X., Ma L., Li Y., Yu J., Duan J. (2025). Surface physicochemical characteristics and wettability regulation mechanism of mango leaves at different growth stages. Sci. Hortic..

[29] Dorr G.J., Wang S., Mayo L.C., McCue S.W., Forster W.A., Hanan J., He X. (2015). Impaction of spray droplets on leaves: Influence of formulation and leaf character on shatter, bounce and adhesion. Exp. Fluids.

[30] Burton Z., Bhushan B. (2006). Surface characterization and adhesion and friction properties of hydrophobic leaf surfaces. Ultramicroscopy.

[31] Ensikat H.J., Mayser M., Barthlott W. (2012). Superhydrophobic and adhesive properties of surfaces: Testing the quality by an elaborated scanning electron microscopy method. Langmuir.

[32] Li J., Cui H., Ma Y., Xun L., Li Z., Yang Z., Lu H. (2020). Orchard Spray Study: A Prediction Model of Droplet Deposition States on Leaf Surfaces. Agronomy.

[33] Jiang Y., Yang Z., Xu X., Shen D., Jiang T., Xie B., Duan J. (2023). Wetting and deposition characteristics of air-assisted spray droplet on large broad-leaved crop canopy. Front. Plant Sci..

[34] Wu S., Liu J., Zhen J., Lei X., Chen Y. (2022). Resistance characteristics of broad-leaf crop canopy in air-assisted spray field and their effects on droplet deposition. Front. Plant Sci..

[35] Huang S., Yan H., Zhang C., Wang G., Acquah S.J., Yu J., Darko R.O. (2020). Modeling evapotranspiration for cucumber plants based on the Shuttleworth-Wallace model in a Venlo-type greenhouse. Agric. Water Manag..

[36] Zhao B., An D., Yan C., Yan H., Kong R., Su J. (2023). Spatiotemporal Variations of Reference Evapotranspiration and Its Climatic Driving Factors in Guangdong, a Humid Subtropical Province of South China. Agronomy.

[37] Yan H., Zhao S., Zhang C., Zhang J., Wang G., Li M., Jiang J. (2024). Calibration and assessment of evapotranspiration methods for cucumber plants in a Venlo-type greenhouse. Irrig. Drain..

[38] Yan H., Acquah S.J., Zhang C., Wang G., Huang S., Zhang H., Wu H. (2019). Energy partitioning of greenhouse cucumber based on the application of Penman-Monteith and Bulk Transfer models. Agric. Water Manag..

[39] Gong C., Li D., Kang C. (2022). Effect of oil-based emulsion on air bubbles in the spray sheet produced through the air-induction nozzle. Pest Manag. Sci..

[40] Gong C., Jia F., Kang C. (2024). Deposition of water and emulsion hollow droplets on hydrophilic and hydrophobic surfaces. Agriculture.

[41] Li H., Travlos I., Qi L., Kanatas P., Wang P. (2019). Optimization of Herbicide Use: Study on Spreading and Evaporation Characteristics of Glyphosate-Organic Silicone Mixture Droplets on Weed Leaves. Agronomy.

[42] Zhou Z., Cao C., Cao L., Zheng L., Xu J., Li F., Huang Q. (2018). Effect of surfactant concentration on the evaporation of droplets on cotton (*Gossypium hirsutum* L.) leaves. Colloids Surf. B Biointerfaces.

[43] Liu D., Pan B., Wang B., Lin Y., Jiang L. (2025). Strategy for the Selection of Tank-Mix Adjuvants to Improve the Wettability of Unmanned Aerial Vehicle-Sprayed Liquids on Citrus Leaf Surfaces. Langmuir.

[44] Gao X., Wang D., Jiang Z., Li X., Chen G. (2022). Effect of Adjuvants on the Wetting Behaviors of Bifenthrin Droplets on Tea Leaves. Appl. Sci..

[45] Arand K., Asmus E., Popp C., Schneider D., Riederer M. (2018). The mode of action of adjuvants—Relevance of physicochemical properties for effects on the foliar application, cuticular permeability, and greenhouse performance of pinoxaden. J. Agric. Food Chem..

[46] Meng Y., Wu Q., Zhou H., Hu H. (2023). How tank-mix adjuvant type and concentration influence the contact angle on wheat leaf surface. PeerJ.

[47] Guo Y., Wang H., Sun W., Sun Y., Xing R., Zhang K., Fang X., Sui B., Xu J. (2025). The Effect of Airflow-Assisted Parameters on Droplet Deposition on Soybean Leaves at the V7 Growth Stage. Agronomy.

[48] Shao C.P., Chen Y.J., Lin J.Z. (2012). Wind induced deformation and vibration of a *Platanus acerifolia* leaf. Acta Mech. Sin..

[49] Tang J., Wang Y., Wang N., Ning X., Lyu K., Sui L., Shi Z. (2020). Swaying Tree Simulation by Slicing Partition. Chin. J. Electron..

[50] Ma Z., Han M., Li Y., Yu S., Chandio F.A. (2020). Comparing kernel damage of different threshing components using high-speed cameras. Int. J. Agric. Biol. Eng..

[51] Zhang B., Chen X., Liang R., Li J., Wang X., Meng H., Kan Z. (2022). Cotton stalk restitution coefficient determination tests based on the binocular high-speed camera technology. Int. J. Agric. Biol. Eng..

[52] Wang G., Dong X., Jia W., Ou M., Yu P., Wu M., Zhang Z., Hu X., Huang Y., Lu F. (2024). Influence of Wind Speed on the Motion Characteristics of Peach Leaves (*Prunus persica*). Agriculture.

[53] Zhang C., Zhou H., Xu L., Ru Y., Ju H., Chen Q. (2022). Measurement of morphological changes of pear leaves in airflow based on high-speed photography. Front. Plant Sci..

[54] Yan C., Niu C., Ma S., Tan H., Xu L. (2022). CFD models as a tool to analyze the deformation behavior of grape leaves under an air-assisted sprayer. Comput. Electron. Agric..

[55] Cao Y., Xi T., Xu L., Qiu W., Guo H., Lv X., Li C. (2022). Computational fluid dynamics simulation experimental verification and analysis of droplets deposition behaviour on vibrating pear leaves. Plant Methods.

[56] Cui H., Wang C., Liu X., Yuan J., Liu Y. (2023). Dynamic simulation of fluid-structure interactions between leaves and airflow during air-assisted spraying: A case study of cotton. Comput. Electron. Agric..

[57] Yazbeck T., Bohrer G., De Roo F., Mauder M., Bakshi B. (2021). Effects of spatial heterogeneity of leaf density and crown spacing of canopy patches on dry deposition rates. Agric. For. Meteorol..

[58] Wang J., Zhang Y., Gu R. (2020). Research Status and Prospects on Plant Canopy Structure Measurement Using Visual Sensors Based on Three-Dimensional Reconstruction. Agriculture.

[59] Liu H., Zhu H. (2016). Evaluation of a laser scanning sensor in detection of complex-shaped targets for variable-rate sprayer development. Trans. ASABE.

[60] Hamed A.M., Sadowski M.J., Nepf H.M., Chamorro L.P. (2017). Impact of height heterogeneity on canopy turbulence. J. Fluid Mech..

[61] Niu Z., Huang T., Xu C., Sun X., Taha M.F., He Y., Qiu Z. (2025). A Novel Approach to Optimize Key Limitations of Azure Kinect DK for Efficient and Precise Leaf Area Measurement. Agricultrue.

[62] Endalew A.M., Debaer C., Rutten N., Vercammen J., Delele M.A., Ramon H., Verboven P. (2010). Modelling pesticide flow and deposition from air-assisted orchard spraying in orchards: A new integrated CFD approach. Agric. For. Meteorol..

[63] Guo S., Li J., Yao W., Zhan Y., Li Y., Shi Y. (2019). Distribution characteristics on droplet deposition of wind field vortex formed by multi-rotor UAV. PLoS ONE.

[64] Zhang H., Qi L., Wan J., Musiu E.M., Zhou J., Lu Z., Wang P. (2022). Numerical simulation of downwash airflow distribution inside tree canopies of an apple orchard from a multirotor unmanned aerial vehicle (UAV) sprayer. Comput. Electron. Agric..

[65] Zhang Z., Lu Y., Zhao Y., Pan Q., Jin K., Xu G., Hu Y. (2023). Ts-yolo: An all-day and lightweight tea canopy shoots detection model. Agronomy.

[66] Zuo Z., Gao S., Peng H., Xue Y., Han L., Ma G., Mao H. (2024). Lightweight Detection of Broccoli Heads in Complex Field Environments Based on LBDC-YOLO. Agronomy.

[67] Zhao Y., Zhang X., Sun J., Yu T., Cai Z., Zhang Z., Mao H. (2024). Low-Cost Lettuce Height Measurement Based on Depth Vision and Lightweight Instance Segmentation Model. Agriculture.

[68] Zhu C., Hao S., Liu C., Wang Y., Jia X., Xu J., Guo S., Huo J., Wang W. (2024). An Efficient Computer Vision-Based Dual-Face Target Precision Variable Spraying Robotic System for Foliar Fertilisers. Agronomy.

[69] Fujimoto A., Satow T., Kishimoto T. (2016). Simulation of spray distribution with boom sprayer considering effect of wind for agricultural cloud computing analysis. Eng. Agric. Environ. Food.

[70] Chen S., Lan Y., Zhou Z., Ouyang F., Wang G., Huang X., Deng X., Cheng S. (2020). Effect of Droplet Size Parameters on Droplet Deposition and Drift of Aerial Spraying by Using Plant Protection UAV. Agronomy.

[71] Liu J., Yuan S., Darko R.O. (2016). Characteristics of water and droplet size distribution from fluidic sprinklers. Irrig. Drain..

[72] Liao J., Hewitt A.J., Wang P., Luo X., Zang Y., Zhou Z., O’Donnell C. (2019). Development of droplet characteristics prediction models for air induction nozzles based on wind tunnel tests. Int. J. Agric. Biol. Eng..

[73] Zhang J., Chen Q., Zhou H., Liu C., Han R., Lv X. (2025). Morphological changes and spray coverage of pear leaves and canopy at different phenological periods during air-assisted spraying. Crop Prot..

[74] Jiang S., Yang S., Xu J., Li W., Zheng Y., Liu X., Tan Y. (2022). Wind field and droplet coverage characteristics of air-assisted sprayer in mango-tree canopies. Pest Manag. Sci..

[75] Meyers T., Tha Paw U K. (1986). Testing of a higher-order closure model for modeling airflow within and above plant canopies. Bound.-Layer Meteorol..

[76] Hong S.W., Zhao L., Zhu H. (2018). CFD simulation of pesticide spray from air-assisted sprayers in an apple orchard: Tree deposition and off-target losses. Atmos. Environ..

[77] Wang S., Ren L., Liu Y., Han Z., Yang Y. (2010). Mechanical characteristics of typical plant leaves. J. Bionic Eng..

[78] Gibson L.J. (2012). The hierarchical structure and mechanics of plant materials. J. R. Soc. Interface.

[79] Xing D., Chen X., Wu Y., Li Z., Khan S. (2021). Changes in elastic modulus, leaf tensity and leaf density during dehydration of detached leaves in two plant species of Moraceae. Chil. J. Agric. Res..

[80] Okonkwo E.G., Daniel-Mkpume C.C., Ude S.N., Onah C.C., Ijomah A.I., Omah A.D. (2019). Chicken feather fiber—African star apple leaves bio-composite: Empirical study of mechanical and morphological properties. Mater. Res. Express.

[81] Cui H., Wang C., Lu F., Liu X., Yuan J. (2023). Dynamic stratified porosity computation from canopy interaction simulation between airflow and leaves. Front. Plant Sci..

[82] Hua L., Jiang Y., Li H., Qin L. (2022). Effects of Different Nozzle Orifice Shapes on Water Droplet Characteristics for Sprinkler Irrigation. Horticulturae.

[83] Knight R.M., Li X., Hocter J.S., Zhang B., Zhao L., Zhu H. (2022). Optimization of Induction Charging of Water Droplets to Develop an Electrostatic Spray Scrubber Intended for Poultry Particulate Matter Mitigation. J. ASABE.

[84] Junping L., Xingye Z., Shouqi Y., Xingfa L. (2018). Droplet motion model and simulation of a complete fluidic sprinkler. Trans. ASABE.

[85] Liao J., Luo X., Wang P., Zhou Z., O’Donnell C.C., Zang Y., Hewitt A.J. (2020). Analysis of the influence of different parameters on droplet characteristics and droplet size classification categories for air induction nozzle. Agronomy.

[86] Jiang Y., Liu J., Li H., Hua L., Yong Y. (2021). Droplet distribution characteristics of impact sprinklers with circular and noncircular nozzles: Effect of nozzle aspect ratios and equivalent diameters. Biosyst. Eng..

[87] Tang Y., Hou C.J., Luo S.M., Lin J.T., Yang Z., Huang W.F. (2018). Effects of operation height and tree shape on droplet deposition in citrus trees using an unmanned aerial vehicle. Comput. Electron. Agric..

[88] Chen R., Li H., Wang J., Guo X. (2021). Analysis of droplet characteristics and kinetic energy distribution for fixed spray plate sprinkler at low working pressure. Trans. ASABE.

[89] Wang Z., Jiang Y., Liu J., Li H., Li H. (2022). Experimental Study on Water Distribution and Droplet Kinetic Energy Intensity from Non-Circular Nozzles with Different Aspect Ratios. Agriculture.

[90] Gao J., Guo Y., Tunio M.H., Chen X., Chen Z. (2023). Design of a high-voltage electrostatic ultrasonic atomization nozzle and its droplet adhesion effects on aeroponically cultivated plant roots. Int. J. Agric. Biol. Eng..

[91] Koc C., Duran H., Gerdan Koc D. (2023). Orchard sprayer design for precision pesticide application. Erwerbs-Obstbau.

[92] Yang Q., Hu Y., Wang Y., Xu B., Zhou C., Adhikari B., Wang B. (2025). Atmosphere-controlled high-voltage electrospray for improving conductivity, flexibility, and antibacterial properties of chitosan films. Food Res. Int..

[93] Zhou L., Zhou B. (2024). Research on Deposition Characteristics of a New Air-Assisted Electrostatic Sprayer. Eur. J. Agric. Food Sci..

[94] Guo Z., Zhang J., Chen L., Wang Z., Wang H., Wang X. (2024). Study on Deposition Characteristics of the Electrostatic Sprayer for Pesticide Application in Greenhouse Tomato Crops. Agriculture.

[95] Liu C., Kang S., Li F., Li S., Du T. (2013). Canopy leaf area index for apple tree using hemispherical photography in arid region. Sci. Hortic..

[96] Zarate-Valdez J.L., Whiting M.L., Lampinen B.D., Metcalf S., Ustin S.L., Brown P.H. (2012). Prediction of leaf area index in almonds by vegetation indexes. Comput. Electron. Agric..

[97] Gu C., Sun J., Li S., Yang S., Zou W., Zhai C. (2025). Deposition Characteristics of Air-Assisted Sprayer Based on Canopy Volume and Leaf Area of Orchard Trees. Plants.

[98] Liu D., Chen L., Tai S., Li Y., Xu C. (2025). Model and experiment of target-specific variable spraying based on canopy volume perception. Crop Prot..

[99] Sun Y., Luo Y., Zhang Q., Xu L., Wang L., Zhang P. (2022). Estimation of Crop Height Distribution for Mature Rice Based on a Moving Surface and 3D Point Cloud Elevation. Agronomy.

[100] Zhao J., Li H., Chen C., Pang Y., Zhu X. (2022). Detection of Water Content in Lettuce Canopies Based on Hyperspectral Imaging Technology under Outdoor Conditions. Agriculture.

[101] Hong S.W., Zhao L., Zhu H. (2018). CFD simulation of airflow inside tree canopies discharged from air-assisted sprayers. Comput. Electron. Agric..

[102] Sun H., Zheng H., Yu H., Qiu W., Cao Y., Lv X., Zhang Z. (2023). CFD simulation of circulating-airflow distribution inside canopy from novel air-assisted sprayer in orchard. J. ASABE.

[103] Cui H., Wang C., Liu X., Yuan J., Liu Y., Song L. (2022). Cotton canopy airflow simulation and velocity attenuation model based upon 3D phenotype and stratified sub-regional porous medium. Comput. Electron. Agric..

[104] Cui H., Wang C., Yu S., Xin Z., Liu X., Yuan J. (2024). Two-stage CFD simulation of droplet deposition on deformed leaves of cotton canopy in air-assisted spraying. Comput. Electron. Agric..

[105] Dietsche L.J., Neubauer A.C. (2009). Computational fluid dynamics model of viscous droplet breakup. Chem. Eng. Sci..

[106] Tavangar S., Hashemabadi S.H., Saberimoghadam A. (2015). CFD simulation for secondary breakup of coal–water slurry drops using OpenFOAM. Fuel Process. Technol..

[107] Zhu W., Zhao N., Jia X., Chen X., Zheng H. (2021). Effect of airflow pressure on the droplet breakup in the shear breakup regime. Phys. Fluids.

[108] He Y., Wu J., Fu H., Sun Z., Fang H., Wang W. (2022). Quantitative Analysis of Droplet Size Distribution in Plant Protection Spray Based on Machine Learning Method. Water.

[109] Paturi U.M.R., Reddy N.S., Cheruku S., Narala S.K.R., Cho K.K., Reddy M.M. (2021). Estimation of coating thickness in electrostatic spray deposition by machine learning and response surface methodology. Surf. Coat. Technol..

[110] Acharya P., Burgers T., Nguyen K.D. (2023). A deep-learning framework for spray pattern segmentation and estimation in agricultural spraying systems. Sci. Rep..

[111] Jiang Y., Li H., Hua L., Zhang D. (2020). Three-dimensional flow breakup characteristics of a circular jet with different nozzle geometries. Biosyst. Eng..

[112] Zhao F., Zhou Z., Hung D., Li X., Xu M. (2024). Flow field reconstruction from spray imaging: A hybrid physics-based and machine learning approach based on two-phase fluorescence particle image velocimetry measurements. Phys. Fluids.

[113] Li W., Yang S., Zhao H., Jiang S., Zheng Y., Liu X., Tan Y. (2024). Deep learning method for leaf-density estimation based on wind-excited audio of fruit-tree canopies. Comput. Electron. Agric..

[114] Violato D., Moore P., Scarano F. (2011). Lagrangian and Eulerian pressure field evaluation of rod-airfoil flow from time-resolved tomographic PIV. Exp. Fluids.

[115] Czyż Z., Karpiński P., Stryczniewicz W. (2020). Measurement of the Flow Field Generated by Multicopter Propellers. Sensors.

[116] Xun L., Gil E. (2024). A novel methodology for water-sensitive papers analysis focusing on the segmentation of overlapping droplets to better characterize deposition pattern. Crop Prot..

[117] Ahmad F., Zhang S., Qiu B., Ma J., Xin H., Qiu W., Ahmed S., Chandio F.A., Khaliq A. (2022). Comparison of Water Sensitive Paper and Glass Strip Sampling Approaches to Access Spray Deposit by UAV Sprayers. Agronomy.

[118] Berrocal E., Kristensson E., Zigan L. (2018). Light sheet fluorescence microscopic imaging for high-resolution visualization of spray dynamics. Int. J. Spray Combust. Dyn..

[119] Wang Y., Jia W., Dai S., Ou M., Dong X., Wang G., Gao B., Tu D. (2025). Analytical Methods for Wind-Driven Dynamic Behavior of Pear Leaves (*Pyrus pyrifolia*). Agriculture.

[120] Hua L., Li H., Jiang Y. (2021). Axis-switching behavior of liquid jets issued from non-circular nozzles under low-intermediate pressure. Appl. Eng. Agric..

[121] Tang P., Chen C. (2022). An Investigation of the Frequency and Duration of a Drive Spoon–Dispersed Water Jet and Its Influence on the Hydraulic Performance of a Large-Volume Irrigation Sprinkler. Agronomy.

[122] Jiang Y., Wang Z., Li H., Wang L. (2024). Optimising the hydraulic performance of a jet impingement sprinkler by varying elevation angle: A Comparative study with a non-impingement sprinkler. Biosyst. Eng..

[123] Ma J., Liu K., Dong X., Huang X., Ahmad F., Qiu B. (2023). Force and motion behaviour of crop leaves during spraying. Biosyst. Eng..

